# Single‐Atom Cu Stabilized on Ultrathin WO_2.72_ Nanowire for Highly Selective and Ultrasensitive ppb‐Level Toluene Detection

**DOI:** 10.1002/advs.202302778

**Published:** 2023-07-13

**Authors:** Peng Wang, Shisong Guo, Zhixiang Hu, Licheng Zhou, Tiankun Li, Shiliang Pu, Hui Mao, Hong Cai, Zhenfeng Zhu, Bingbing Chen, Hua‐Yao Li, Huan Liu

**Affiliations:** ^1^ School of Integrated Circuits Wuhan National Laboratory for Optoelectronics Optics Valley Laboratory Huazhong University of Science and Technology 1037 Luoyu Road Wuhan Hubei 430074 P. R. China; ^2^ Wenzhou Key Laboratory of Optoelectronic Materials and Devices Application Wenzhou Advanced Manufacturing Institute of HUST 1085 Meiquan Road Wenzhou Zhejiang 325035 P. R. China; ^3^ Hikvision Research Institute 555 Qianmo Road Hangzhou Zhejiang 310051 P. R. China; ^4^ School of Energy Science and Engineering Nanjing Tech University Nanjing Jiangsu 211816 P. R. China

**Keywords:** chemiresistive gas sensors, Cu single‐atom catalysts, high selectivity, ppb level, toluene

## Abstract

Various catalysts are developed to improve the performance of metal oxide semiconductor gas sensors, but achieving high selectivity and response intensity in chemiresistive gas sensors (CGSs) remains a significant challenge. In this study, an in situ‐annealing approach to synthesize Cu catalytic sites on ultrathin WO_2.72_ nanowires for detecting toluene at ultralow concentrations (*R*_a_/*R*_g_ = 1.9 at 10 ppb) with high selectivity is developed. Experimental and molecular dynamic studies reveal that the Cu single atoms (SAs) act as active sites, promoting the oxidation of toluene and increasing the affinity of Cu single‐atom catalysts (SACs)‐containing sensing materials for toluene while weakening the association with carbon dioxide or water vapor. Density functional theory studies show that the selective binding of toluene to Cu SAs is due to the favorable binding sites provided by Cu SAs for toluene molecules over other gaseous species, which aids the adsorption of toluene on WO_2.72_ nanowires. This study demonstrates the successful atomic‐level interface regulation engineering of WO_2.72_ nanowire‐supported Cu SAs, providing a potential strategy for the development of highly active and durable CGSs.

## Graphical abstract

A cost‐effective approach for depositing Cu single atoms (SAs) onto ultrathin WO_2.72_ nanowires to create an efficient toluene gas sensor is presented. The resulting Cu SA/WO_2.72_‐4% sensor demonstrates exceptional response intensity (*R*_a_/*R*_g_ = 1.9 at 10 ppb), ultrafast response (15 s), high selectivity, and excellent reversibility, all at an extremely low actual detection concentration (10 ppb).

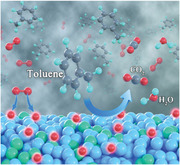

## 1 Introduction

Chemiresistive gas sensors (CGSs) are becoming increasingly important for ensuring public safety, environmental monitoring, and healthcare diagnosis due to their low cost, easy miniaturization, and environmental friendliness.^[^ ^1^, ^2^ ^]^ However, their widespread use is hindered by the challenge of developing efficient and cost‐effective catalysts for the redox reactions of gas molecules over the sensitive layers.^[^ ^3^ ^]^ Currently, transition‐group‐metal‐based materials are the most effective catalysts for meeting the high selectivity and response intensity requirements of practical applications. However, their high cost and tendency to agglomerate into clusters limit their application. To overcome these challenges, researchers are exploring the use of single transition metal atoms strongly anchored on support materials to combine the benefits of both homo‐ and heterogenous catalysis. This approach has the potential to address the concerns associated with transition‐group‐metal‐based materials and pave the way for the development of more efficient and cost‐effective CGSs.^[^ ^4^, ^5^ ^]^

Single‐atom catalysts (SACs) offer homogeneity of active sites and consistency of the local coordination environment, providing exceptional selectivity and high efficiency for a variety of catalytic reactions compared to traditional cluster catalysts or nanoparticles.^[^ ^6^, ^7^, ^8^ ^]^ Most importantly, the SACs possess the maximum atom utilization efficiency, ideally reaching 100%.^[^ ^9^ ^]^ Hence, it has been widely explored for different reactions, including carbon dioxide reduction reaction, carbon monoxide oxidation reaction, nitric oxide reduction reaction, gas‐sensing reaction, and other redox reactions.^[^ ^10^, ^11^ ^]^ In the field of gas sensors, Zhang et al.^[^ ^11^ ^]^ reported the prospects for the creation of new gas‐sensing materials with emerging SACs and versatile supports. Kim et al.^[^ ^12^ ^]^ demonstrated a formaldehyde gas sensor based on Pt SACs. The supported Pt single atoms (SAs), established by the 1D nanoheterostructure approach, exhibited high response intensity and selectivity toward the detection of formaldehyde. Duan et al.^[^ ^13^ ^]^ employed Pd SAs to functionalize the surface of In_2_O_3_ nanosheets (Pd SACs/In_2_O_3_), resulting in a hydrogen sulfide sensor with higher response intensity and selectivity. Li et al.^[^ ^14^ ^]^ rationally decorated Pt SAs on the surface of porous γ‐Fe_2_O_3_ nanoparticles via a simple sol‐thermal reaction for ethanol detection. The Pt SACs/γ‐Fe_2_O_3_ displayed a higher adsorption capacity for ethanol, and the gas‐sensing performance was improved. Thus, we believe that developing practical gas‐sensing materials for toxic gas detection requires the use of SACs. However, despite the potential benefits of SACs in gas sensing, research in the field is still in its initial stages, and the detailed gas‐sensing mechanism through SACs activation remains unclear. Moreover, while SAC research in gas sensing primarily focuses on noble metals such as Pt and Pd, etc., reports on nonnoble metals such as Cu, Ni, and Co SACs, etc., are still scarce. Further investigation is necessary to fully realize the potential of SACs in gas sensing and to expand the range of available SACs for practical applications.

Considering the atom economy and energy consumption, the creation of SACs with high performance is highly desirable. Copper, as a transition metal, is regarded as a good candidate to serve as a promising catalyst due to their exceptional properties, including low‐cost, excellent catalytic activity, and stability.^[^ ^15^, ^16^ ^]^ Recently, Zhao et al.^[^ ^15^ ^]^ successfully synthesized a copper‐based catalyst with isolated active copper sites to realize the CO_2_ hydrogenation reaction to CH_3_OH. The Cu SACs/ZrO_2_ displayed a higher turnover frequency for CH_3_OH and 100% CH_3_OH selectivity. Xie et al.^[^ ^16^ ^]^ constructed a photocatalyst based on Cu‐N4 sites anchored phosphorus‐modulated carbon nitride (Cu SACs/PCN). The Cu SACs/PCN exhibited a high C_2_H_4_ selectivity of 53.2% with a yielding rate of 30.51 µmol g^−1^. Inspired by their studies, we believe that the highly consistent catalytic specificity of Cu SAs can avoid various occurrences of side reactions, improving exclusively the gas‐sensing reaction on the sensitive layer surface of the CGS.

Toluene is a volatile organic compound (VOC) commonly used in various applications such as furniture, rubber cement, paint, and chemical reagents.^[^ ^17^ ^]^ However, exposure to toluene can have serious effects on the skin and central nervous system, and it is classified as a carcinogen by the World Health Organization, with indoor concentrations recommended to be kept below 70 ppb.^[^ ^18^, ^19^ ^]^ Therefore, there is a need to develop highly selective and ultrasensitive gas sensors capable of detecting sub‐parts per million (ppb)‐level toluene.

In this study, we design single‐atom Cu sites anchored on ultrathin WO_2.72_ nanowires (Cu SA/WO_2.72_) as the gas‐sensing material for toluene detection. The Cu SA/WO_2.72_‐4% (wherein 4% represents the theoretical weight percentage of CuCl_2_ to WCl_6_, i.e., 4 wt%) sensor exhibits an extremely low actual detection concentration (10 ppb), ultrasensitive response (*R*_a_/*R*_g_ = 1.9 at 10 ppb), the ultrafast response time (15 s), and excellent reversibility. The nice response for toluene detection is attributed to the dynamic surface modulation of Cu SAs affecting the gas molecule adsorption energy. In‐depth experimental and various techniques, including density functional theory (DFT) calculations, molecular dynamics (MD) simulations, and in situ IR spectroscopy, provide atomic‐level insights into the gas‐sensing mechanism of Cu SA/WO_2.72_‐based sensors. Overall, our results demonstrate the successful atomic‐level interface regulation engineering of WO_2.72_ nanowire‐supported Cu SACs for toluene detection, and highlight the potential of this design strategy for developing highly active and durable CGSs.

## 2 Results and Discussion

### 2.1 Synthesis and Characterizations of the Ultrathin Cu SA/WO_2.72_ Nanowires

**Figure 1:**
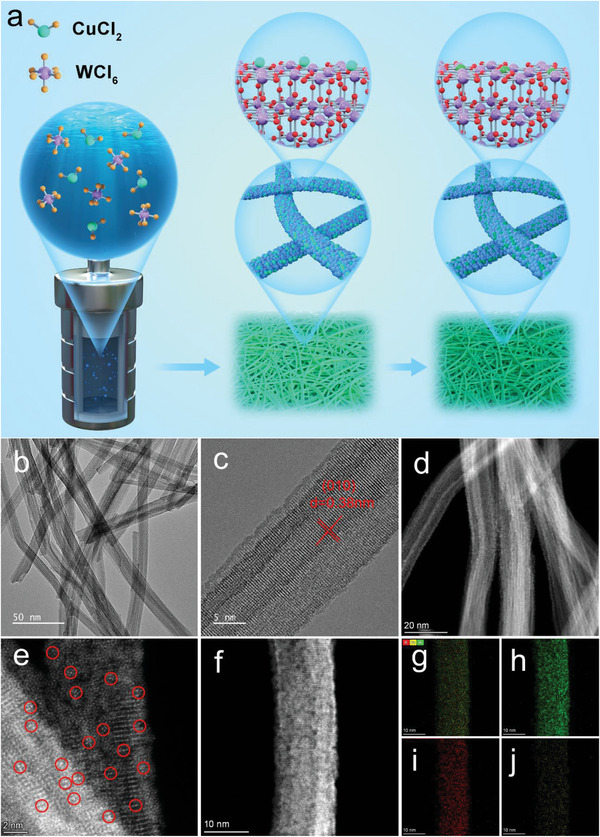
a) Schematic illustration for the synthesis of Cu SA‐anchored ultrathin WO_2.72_ nanowires (Cu SA/WO_2.72_). Characterization of Cu SA/WO_2.72_‐4%. b) TEM image, c) HRTEM image, d,e) AC HAADF‐STEM image, and f) AC HAADF‐STEM image and g–j) corresponding EDX elemental mapping.

The Cu SACs on ultrathin WO_2.72_ nanowires have been successfully synthesized via a preassembly step in an ethanol solution followed by an annealing process (**Figure** **1**a). In control experiments, Cu nanoparticles (NPs) supported by WO_2.72_ nanowires and Cu SA/WO_2.72_ with different Cu loadings are prepared (for a detailed description, see the Supporting Information). As shown in Figure S3a,b, Supporting Information, the as‐synthesized WO_2.72_ appears the 1D nanowires with a uniform diameter. Energy‐dispersive spectrometry (EDS) spectrum shows that the main elements are W and O (Figure S3d–g, Supporting Information). Compared with​ WO_2.72_ without Cu, the transmission electron microscope (TEM) image shows that the 1D morphology is well maintained in Cu SA/WO_2.72_‐4% sample (Figure 1b). Interestingly, the local magnification TEM image (Figure S4, Supporting Information) clearly reveals that Cu SA/WO_2.72_‐4% is a 1D nanowire structure formed by interconnected thinner nanowires.^[^ ^20^ ^]^ It has a Brunauer–Emmett–Teller specific surface area of 98.72 m^2^ g^−1^ and a hierarchically nanoporous structure that can facilitate the entry and the release of gas molecules (Figures S5 and S6 and Table S1, Supporting Information). A high‐resolution TEM (HRTEM) image (Figure 1c) verifies clear lattice fringes with a crystal interplanar spacing of about 0.38 nm, which corresponds to the (010) crystal plane.^[^ ^21^ ^]^ Meanwhile, the EDS element mapping of Cu SA/WO_2.72_‐4% indicates the existence and uniform distribution of W, O, and Cu elements (Figure S7, Supporting Information). To further determine the atomic structure of Cu SA/WO_2.72_‐4% sample, annular dark‐field imaging has been conducted on an aberration‐corrected scanning transmission electron microscope (STEM) system (Figure 1d–j). The aberration‐corrected high‐angle annular dark‐field STEM (AC HAADF‐STEM) images show individual Cu atoms uniformly distributed over the WO_2.72_ nanowires, and no Cu nanoparticles or clusters could be found, in good accordance with the X‐ray diffraction (XRD) analysis (Figure S11, Supporting Information). Further, the AC HAADF‐STEM image and the energy‐dispersive X‐ray spectroscopy (EDX) elemental mapping reveal the even distribution of the Cu atoms throughout the whole nanowires. By contrast, Cu NPs are observed on reference Cu NPs/WO_2.72_ sample (Figure S8, Supporting Information). To note, the Cu SA/WO_2.72_ samples with different Cu loadings prepared in a similar method are also characterized to be free of Cu NPs (Figures S9 and S10, Supporting Information).

Powder XRD is applied for crystallographic structure characterization of the as‐synthesized samples, as shown in Figure S11, Supporting Information. For pristine WO_2.72_, the two narrow peaks match well with the (010) and (020) reflections of the monoclinic phase WO_2.72_. Meanwhile, all other reflections are much broaden, indicating a preferred orientation along the [010] direction, which will be further demonstrated by the direct observation of the HRTEM image (Figure S3c, Supporting Information).^[^ ^22^ ^]^ Interestingly, after being loaded with Cu atoms, the characteristic peaks of WO_2.72_ phase remain unchanged, i.e., the introduction of Cu atoms does not affect crystal structure. Most importantly, no observable Cu NPs peaks could be detected, which excludes the presence of large copper clusters. Raman spectra (Figure S12, Supporting Information) show that the bands around 256 and 325 cm^−1^ are ascribed to the O─W─O bending mode, and the bands at about 703 and 804 cm^−1^ are attributable to the W─O stretching mode.^[^ ^23^, ^24^ ^]^ It is worth noting that, compared with pristine WO_2.72_, the positions of the peaks around 256, 325, and 703 cm^−1^ shift to a high wavenumber after being loaded with Cu atoms, representing a blue shift.^[^ ^25^ ^]^ Combined with electron paramagnetic resonance and UV‐visible‐near‐infrared absorption spectra (Figures S13 and S14, Supporting Information), the introduction of Cu atoms increases the number of oxygen vacancies, i.e., the local atomic structure disorders/defects.^[^ ^26^, ^27^, ^28^ ^]^ The corresponding Fourier transform infrared spectra results indicate the successful introduction of Cu atoms and the maintenance of WO_2.72_ crystal structure (Figure S15, Supporting Information).

**Figure 2:**
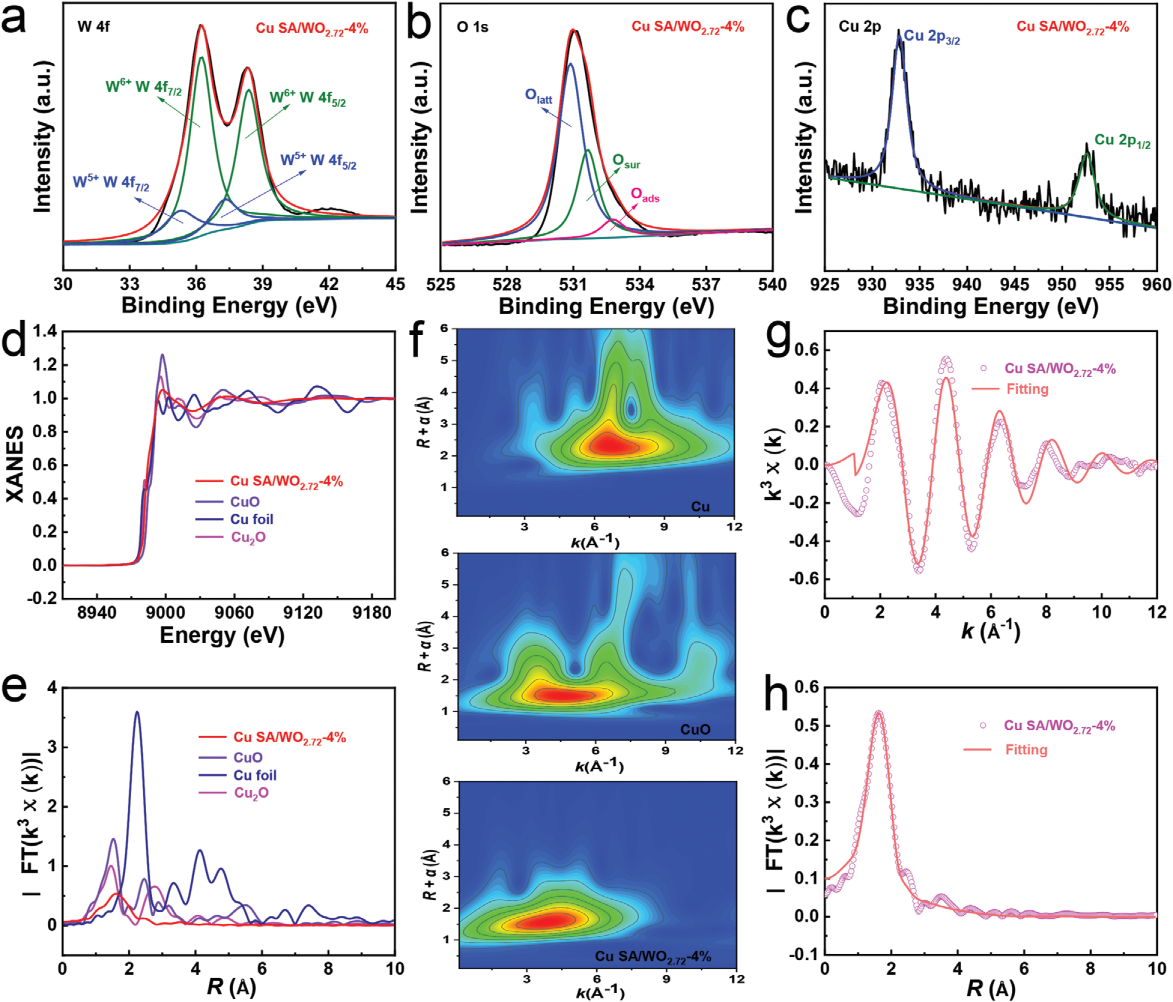
The high‐resolution XPS spectra of a) W 4f peak, b) O 1s peak, and c) Cu 2p peak for Cu SA/WO_2.72_‐4%. d) XANES spectra and e) FT‐EXAFS spectra of the Cu K edge for Cu foil, CuO, Cu_2_O, and Cu SA/WO_2.72_‐4%. f) WT‐EXAFS plots of Cu SA/WO_2.72_‐4%, CuO, and Cu foil. Fourier‐transformed magnitudes of Cu K‐edge EXAFS spectra in g) K space and h) R space for Cu SA/WO_2.72_‐4%.

The surface elemental composition and chemical state of the as‐synthesized samples are determined by X‐ray photoelectron spectroscopy (XPS), revealing the core level peaks of W 4f, O 1s, and Cu 2p. The W 4f spectra located at the binding energies of 35.7 and 37.8 eV are attributed to W^6+^, and the other two peaks centered at the binding energies of 34.8 and 37.1 eV are characteristic of W^5+^ (**Figure** **2**a and Figure S16a, Supporting Information).^[^ ^29^ ^]^ The asymmetric peaks of O 1s could be well fitted into three peaks (530.2, 531.6, and 532.7 eV), corresponding to the lattice oxygen species (O_latt_), the surface hydroxyls formed upon dissociative adsorbed water molecules (O_sur_), and the irreversibly adsorbed molecular mode (O_ads_), respectively (Figure 2b and Figure S16b, Supporting Information).^[^ ^30^ ^]^ It is well accepted that the main signals centered at 932.2 and 952.1 eV correspond to Cu^1+^, and the other signals located at 934.7 and 954.5 eV correspond to Cu^2+^.^[^ ^31^, ^32^ ^]^ As shown in Figure 2c and Figure S16c, Supporting Information, the XPS spectra in the Cu 2p region of Cu SA/WO_2.72_ indicate that the valence of Cu atom is between +1 and +2. To further investigate the local atomic structure of the Cu atom center in the Cu SA/WO_2.72_‐4% sample, we conduct the X‐ray absorption near‐edge structure (XANES) and extended X‐ray absorption fine structure (EXAFS) analyses at the Cu K‐edge. The XANES edge profile of the Cu SA/WO_2.72_‐4% is located between those of Cu_2_O and CuO (Figure 2d), implying that the oxidation state of the atomic Cu species is higher than Cu^+^ and lower than Cu^2+^, which agrees well with the results of XPS analysis.^[^ ^33^ ^]^ The Fourier‐transformed (FT) k^3^‐weighted (FT‐EXAFS) curve of Cu SA/WO_2.72_‐4% only shows a prominent peak at ≈1.6 Å, which is mainly assigned to Cu─O contribution (Figure 2e). In addition, no signals for Cu─Cu bonding are observed in Cu SA/WO_2.72_‐4%, confirming that the Cu species should exist in the form of isolated atoms without any aggregation in the form of Cu particles or clusters (Figure S17, Supporting Information), which is consistent with the AC HAADF‐STEM observations. Wavelet transform (WT) at the Cu K‐edge EXAFS analysis is also conducted to reveal the atomic dispersion of Cu in Cu SA/WO_2.72_‐4% sample. The WT contour plots of the Cu SA/WO_2.72_‐4% only show one intensity maximum at about 3.9 Å^−1^, associated with the Cu─O bonding, which is significantly different from that of the Cu foil, CuO, and Cu_2_O (Figure 2f and Figure S18, Supporting Information). More remarkably, no intensity maximum ascribed to the Cu─Cu contribution is detected.^[^ ^34^ ^]^ As displayed in Figure 2g,h, the EXAFS fitting results show that the average coordination number for the isolated Cu sites within the Cu SA/WO_2.72_‐4% sample is 3.6 with a bond length of 1.96 Å (Table S2, Supporting Information).

### 2.2 Investigation of Gas‐Sensing Performance

**Figure 3:**
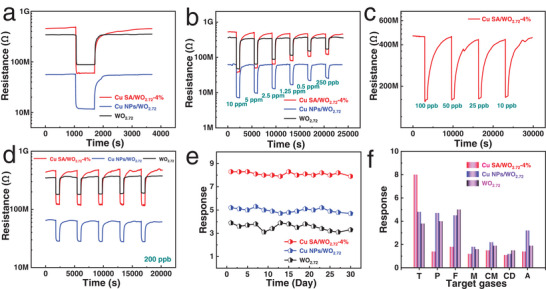
a) Dynamic resistance transition of Cu SA/WO_2.72_‐4% sensor under 2.5 ppm toluene exposure and recovery in comparison with reference samples (pristine WO_2.72_ and Cu NPs/WO_2.72_). b) Toluene gas‐sensing results of Cu SA/WO_2.72_‐4% sensor compared with reference samples (pristine WO_2.72_ and Cu NPs/WO_2.72_). c) Dynamic resistance transition of Cu SA/WO_2.72_‐4% sensor to 10–100 ppb toluene. d) Cyclic stability of Cu SA/WO_2.72_‐4% sensor through five cycles of 200 ppb toluene exposure in comparison with reference samples (pristine WO_2.72_ and Cu NPs/WO_2.72_). e) Long‐term stability of Cu SA/WO_2.72_‐4% sensor under 2.5 ppm toluene exposure in comparison with reference samples (pristine WO_2.72_ and Cu NPs/WO_2.72_). f) Selectivity of Cu SA/WO_2.72_‐4% sensor toward seven different gas species (2.5 ppm) (T is toluene, P is paraxylene, F is formaldehyde, M is methane, CM is carbon monoxide, CD is carbon dioxide, and A is ammonia) compared with reference samples (pristine WO_2.72_ and Cu NPs/WO_2.72_).

To demonstrate the superiority of SACs in terms of gas sensor, it is applied as a chemiresistive gas‐sensing system. The alumina substrate with gold electrodes is used for chemical gas‐sensing tests. The gas‐sensing performance of the Cu SA/WO_2.72_‐4% and the reference samples is examined (Figures S20 and S21, Supporting Information). **Figure** **3**a compares the dynamic resistance transition of pristine WO_2.72_, Cu SA/WO_2.72_‐4%, and Cu NPs/WO_2.72_ sensors toward 2.5 ppm toluene at 160 °C, which is the optimum operating temperature for all prepared sensors. By observing dynamic resistance transitions of pristine WO_2.72_ and Cu SA/WO_2.72_‐4% sensors upon toluene exposure, which reveals an increase in the baseline resistance for Cu SA/WO_2.72_‐4% (≈479 MΩ) over that of pristine WO_2.72_ (≈346 MΩ). We believe that an increase in the chemisorbed oxygen species thickens the electron depletion layer of Cu SA/WO_2.72_‐4%, thereby increasing the baseline resistance of gas sensor.^[^ ^35^ ^]^ Considering the perspective of practical application, we further analyze the response and recovery time of the sensor. As shown in Figure S22a, Supporting Information, the response times are attained for the pristine WO_2.72_ sensor (54 s) and Cu NPs/WO_2.72_ sensor (21 s), while 15 s for the Cu SA/WO_2.72_‐4% sensor, suggesting 3.6 and 1.4 times higher response rate, respectively. This clearly demonstrates that the introduction of Cu SAs could improve the response speed. The corresponding recovery times are determined to be 202, 331, and 307 s, respectively (Figure S22b, Supporting Information). Besides, the response values of the pristine WO_2.72_ and Cu NPs/WO_2.72_ sensors are 3.8 and 4.8, respectively (Figure S22c, Supporting Information). However, the Cu SA/WO_2.72_‐4% sensor exhibits a remarkable response of 8.1, reaching the advanced level of recently reported excellent toluene gas sensors (Table S5, Supporting Information). Then, the gas‐sensing properties of the pristine WO_2.72_, Cu NPs/WO_2.72_, and Cu SA/WO_2.72_‐4% sensors upon exposure to toluene gas over a wide range of concentrations from 250 to 10 000 ppb are evaluated (Figure 3b). The Cu SA/WO_2.72_‐4% sensor not only shows higher response values when exposed to different concentrations of toluene gas but also exhibits a good reversible curve after six periodic circulations. Meanwhile, the response values of the Cu SA/WO_2.72_‐4% sensor always exhibits the fastest rising trend (Figure S23, Supporting Information), ​indicating the potential for a higher concentration of toluene detection. As displayed in Figure S24, Supporting Information, the response–recovery curve measurements are conducted on pristine WO_2.72_, Cu NPs/WO_2.72_, and Cu SA/WO_2.72_‐4% sensors exposed to higher concentrations of toluene (25–400 ppm). The research findings demonstrate that these sensors are capable of monitoring high‐concentration toluene gas in an environment. More importantly, the Cu SA/WO_2.72_‐4% sensor surprisingly shows response values of 1.9, 2.3, 2.6, and 3.2 to 10, 25, 50, and 100 ppb toluene, respectively (Figure 3c and Figure S25, Supporting Information). The theoretical limit of detection (LOD) of the sensor is described in detail, as shown in Figures S26–S28, Supporting Information. It could be concluded that the LODs are 2250, 545, and 333 ppt for the pristine WO_2.72_, Cu NPs/WO_2.72_, and Cu SA/WO_2.72_‐4% sensors, respectively. Compared to metal oxide semiconductor (MOS) gas sensors reported before, as shown in Table S5, Supporting Information, the Cu SA/WO_2.72_‐4% sensor shows either a lower actual detection limit than most other toluene sensors or a higher response intensity at low operating temperature. Moreover, we demonstrate the actual detection of ppb‐level toluene (*R*_a_/*R*_g_ = 1.9 at 10 ppb), which could be comparable to the high‐level work reported recently.

To investigate the reproducibility of toluene detection, as shown in Figure 3d, the dynamic resistance transitions of pristine WO_2.72_, Cu NPs/WO_2.72_, and Cu SA/WO_2.72_‐4% sensors under the cyclic exposure to 200 ppb toluene are plotted. The Cu SA/WO_2.72_‐4% sensor exhibits a stable response intensity of 3.8 with a standard deviation of 0.01 throughout the five cycles, attesting to its high operational stability. Similarly, the pristine WO_2.72_ and Cu NPs/WO_2.72_ sensors also show high stable response intensity, with standard deviations of 0.04 and 0.03, respectively (for a detailed explanation regarding the standard deviation of sensor, see Supporting Information). Furthermore, we further investigate the dynamic resistance transitions of the aforementioned sensors under the cyclic exposure to 250 ppm toluene (Figure S29, Supporting Information), demonstrating stable cyclic performance without any noticeable attenuation. More remarkably, the long‐term stability of Cu SA/WO_2.72_‐4% sensor is excellent (Figure 3e), which is as we expect when we observe that the Cu SAs are anchored on the ultrathin WO_2.72_ nanowires. As a result, the attenuation ratio of the sensor is only 4.8% after 30 days of testing. In sharp contrast, the pristine WO_2.72_ sensor shows an attenuation ratio of 15.2%. Besides, the attenuation ratio is 9.6% for the Cu NPs/WO_2.72_ sensor after 30 days of testing (for a detailed explanation regarding the attenuation ratio of sensor, see Supporting Information). For such exceptional long‐term stability of sensor, Cu SA/WO_2.72_‐4% in particular, it may be due to the stability of the Cu SACs. To verify this hypothesis, we further conduct corresponding characterization analysis on sensitive material operated under 160 °C for 30 days. There is no apparent alteration in the morphology and chemical composition of the Cu SA catalytic center in the Cu SA/WO_2.72_‐4% after 30 days of toluene gas‐sensing test, further demonstrating its outstanding stability (Figure S30, Supporting Information). In addition, the response curves of the pristine WO_2.72_, Cu SA/WO_2.72_‐4%, and Cu NPs/WO_2.72_ sensors under different humidity conditions are given, as shown in Figure S31, Supporting Information. The response values decrease as the relative humidity (RH) increases from 10% to 70%, indicating that there is a humidity effect. However, the above‐mentioned sensors are still functioning well in a humidity environment, especially at high RH.

Figure 3f compares the selectivity for pristine WO_2.72_, Cu SA/WO_2.72_‐4%, and Cu NPs/WO_2.72_ sensors. For the pristine WO_2.72_ sensor, we observe that the response for toluene approximates to those of other VOCs, such as formaldehyde and paraxylene. When Cu NPs are introduced into the WO_2.72_ surface, the response behavior of the sensor varies, showing an increased response to toluene, but also a higher response to ammonia, paraxylene, and formaldehyde, implying that the selectivity of the Cu NPs/WO_2.72_ sensor is not optimal. However, the Cu SA/WO_2.72_‐4% sensor exhibits remarkable selectivity toward toluene while exhibiting negligible cross‐response toward formaldehyde, paraxylene, methane, carbon monoxide, carbon dioxide, and ammonia. These results indicate that in addition to oxygen spill‐over effect,^[^ ^5^ ^]^ Cu SAs also provide more favorable binding sites for toluene molecules than other gaseous species, contributing to the adsorption of toluene on WO_2.72_ nanowires. Thus, more toluene molecules are oxidized by reactive oxygen species, leading to enhanced gas‐sensing performance, especially for distinguishing toluene gas in a complex atmosphere. In order to understand deeply the reason for such selectivity variation, adsorption behaviors of different gas molecules on pristine WO_2.72_, Cu SA/WO_2.72_, and Cu NPs/WO_2.72_ ​are explored by performing DFT calculation. As shown in Figure S34, Supporting Information, the adsorption energies of Cu SA/WO_2.72_ to various gases (toluene, formaldehyde, paraxylene, methane, carbon monoxide, carbon dioxide, and ammonia) are studied. Noticeably, the adsorption energy of toluene is −1.659 eV, much lower than other gases. The result indicates that the adsorption capacity for toluene on Cu SA/WO_2.72_ is much stronger than other interfering gases, which may be a major reason for the excellent selectivity toward toluene gas. Furthermore, the adsorption energies of different gas molecules on pristine WO_2.72_ and Cu NPs/WO_2.72_ are calculated (Figures S35 and S36, Supporting Information). For the pristine WO_2.72_ crystal structure, the adsorption energy of toluene is similar to that of other gas molecules, such as formaldehyde and paraxylene. For the Cu NPs/WO_2.72_, we observe no significant differentiation in the adsorption energies of toluene, ammonia, methane, and paraxylene. These findings testify that the introduction of different types of Cu species leads to the distinction in adsorption capacity toward various gas molecules, which may be the cause of the selectivity variation.

### 2.3 Insight into the Origin of High Gas‐Sensing Performance

Today, the gas‐sensing mechanism of MOSs has been extensively reported, i.e., the redox reactions between active oxygen species and target gas molecules. In an ambient atmosphere, the adsorbed oxygen molecules on the surface of sensing layer attract electrons from the conduction band to form charged oxygen species ($O_{2}^{-}$, O^−^, and O^2 −^ ).^[^ ^36^, ^37^ ^]^ Then, toluene gas molecules react with the active oxygen species to promote the release of the captured electrons back to the conduction band, thus decreasing the resistance of the sensor as observed. As reported, oxygen vacancy, specific surface area, and micromorphology play important roles in toluene gas detection.^[^ ^38^ ^]^ As shown in Figure S5 and Table S1, Supporting Information, the Cu SAs anchored on the ultrathin WO_2.72_ nanowires increase the specific surface area to provide more active sites for toluene gas molecules (Figures S37 and S38, Supporting Information). Meanwhile, more oxygen vacancies (Figure S13, Supporting Information) facilitate the enhancement of toluene detection. In addition, we acquire the equilibrium constant (*K*_F_) based on the Freundlich isothermal model for toluene response (for a detailed explanation regarding the isothermal response model of sensor, see Supporting Information). The results show that the Cu SA/WO_2.72_‐based sensor features up to 2.7‐fold improvements in the *K*_F_, compared to the pristine WO_2.72_ sensor. Significantly, the larger the *K*_F_ value, the better the sensor performance.^[^ ^39^, ^40^ ^]^ Consequently, as has been discussed before, the Cu SAs anchored on the ultrathin WO_2.72_ nanowires are more beneficial for the toluene gas‐sensing performance, especially for the exceptional long‐term stability and high response intensity.

**Figure 4:**
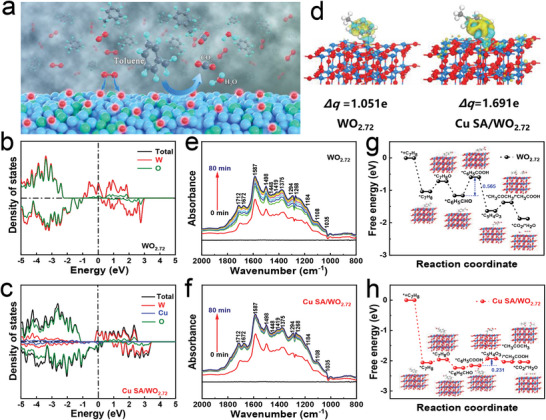
a) Schematic illustration for toluene sensing mechanism on Cu SA/WO_2.72_‐based sensor. The DOS of b) pristine WO_2.72_ and c) Cu SA/WO_2.72_ samples. d) The differential charge density and corresponding Bader charge (Δ*q*) of toluene gas molecules adsorbed on pristine WO_2.72_ and Cu SA/WO_2.72_ samples. In situ DRIFTS spectra (800–2000 cm^−1^) for the oxidation of toluene over e) pristine WO_2.72_ and f) Cu SA/WO_2.72_ samples at different reaction time. Energy paths of intermediates during oxidation of toluene to carbon dioxide and water vapor on g) pristine WO_2.72_ and h) Cu SA/WO_2.72_ samples.

It is well accepted that, when the size of the transition metal catalyst is reduced to the atomic scale, the electronic properties of the metal catalyst will change dramatically, which may lead to variation in the gas‐sensing mechanism. Here, we propose the toluene sensing and conversion mechanism on Cu SA/WO_2.72_‐based sensor, as shown in **Figure** **4**a and Figure S41, Supporting Information. Thus, to verify the gas‐sensing mechanism of sensing material regulation at a single atomic scale, we implement DFT calculation, in situ IR spectroscopy, and MD simulation.

The DFT calculations study the origin of the remarkable performance over Cu SA/WO_2.72_ for toluene detection. Figure 4b,c displays the density of states (DOS) of pristine WO_2.72_ and Cu SA/WO_2.72_ samples. As revealed, the most prominent characteristic in Cu SA/WO_2.72_ sample is that the electrons show continuously occupied states at the Fermi level, in sharp contrast to the WO_2.72_ without Cu SAs. Interestingly, the conduction band of Cu SA/WO_2.72_ is much closer to the Fermi level than that of pristine WO_2.72_, demonstrating the better charge transfer capability of Cu SA/WO_2.72_.^[^ ^41^ ^]^ Meanwhile, the work functions of pristine WO_2.72_ and Cu SA/WO_2.72_ sample films are measured by the Kelvin Probe test system in an air atmosphere, resulting in values of 5.24 and 4.88 eV, respectively. The functionalization of Cu SAs leads to a decrease in the work function of pristine WO_2.72_ by 0.36 eV, indicating an enhanced availability of reactive electrons from WO_2.72_ in the presence of Cu SAs.^[^ ^42^ ^]^ Furthermore, as confirmed by the differential charge densities over the toluene‐adsorbed structural models, higher concentrations of electrons are transferred to the near‐surface region of the Cu SA/WO_2.72_ sample (Figure 4d). The results verify that the introduction of Cu SAs could further enhance electrical conductivity and accelerate electron transport, which is beneficial to the improvement of gas‐sensing properties.

The in situ IR spectroscopy is performed to study the gas–solid interfacial molecular evolution ​in the toluene oxidation process. As shown in Figure 4e,f and Figure S42, Supporting Information, with the increase in reaction time, the peak intensity of IR spectra increases gradually. Furthermore, by comparing the IR spectra with a reaction time of 80 min, we find that the peak intensity of Cu SA/WO_2.72_ is slightly higher, indicating that the gas‐sensing reaction is more intense than that of pristine WO_2.72_ (Figure S43, Supporting Information). Inspired by previous studies,^[^ ^43^, ^44^ ^]^ these results indicate that adsorbed toluene is sequentially oxidized by the surface‐active oxygen to generate gas‐sensing intermediates, such as benzyl alcohol, benzaldehyde, benzoic acid, itaconic anhydride, acetone, and acetic acid. From this, the oxidation of toluene involves six successive steps in addition to the adsorption and desorption steps, in which oxidation occurs to form carbon dioxide and water vapor as the final products (*C_7_H_8_ → *C_7_H_7_ − OH → *C_6_H_5_ − CHO → *C_6_H_5_ − COOH → *C_5_H_4_O_3_ → *CH_3_COCH_3_ + *CH_3_COOH → *CO_2_ + *H_2_O).^[^ ^45^ ^]^ Thus, the oxidation steps, the Gibbs free energy profiles and the reaction intermediate geometries of toluene, are investigated in detail by the DFT calculation. Figure 4g,h displays the relative energy profiles of the reaction pathway over the pristine WO_2.72_ and Cu SA/WO_2.72_ samples. It could be seen that the process of toluene from the gaseous state to the adsorbed state is strongly exothermic.^[^ ^46^ ^]^ This process provides adequate energy for the above‐mentioned intermediates’ activation, resulting in the intermediates reacting with active oxygen species is a spontaneous process, which is consistent with the thermodynamics study results (for a detailed explanation regarding the thermodynamics of sensor, see Supporting Information). It is worth noting that, the rate‐determining step for the overall oxidation process on the pristine WO_2.72_ surface is the third oxidation step (*C_6_H_5_ − CHO → *C_6_H_5_ − COOH) (Figure 4g), while on the Cu SA/WO_2.72_ surface is the fourth oxidation step (*C_6_H_5_ − COOH → *C_5_H_4_O_3_) (Figure 4h).^[^ ^47^, ^48^ ^]^ Meanwhile, the energy barriers for the key steps of toluene oxidation on the pristine WO_2.72_ and Cu SA/WO_2.72_ are + 0.565 and + 0.231 eV, respectively, which is naturally easier to overcome on the Cu SA/WO_2.72_ surface. The above analysis shows that for pristine WO_2.72_, the overall barrier of toluene oxidation to carbon dioxide and water vapor is higher than that over Cu SA/WO_2.72_, as a result, the Cu SA/WO_2.72_‐based sensors show superior toluene gas‐sensing performance than the pristine WO_2.72_. Moreover, the charge transport activation energy (*E*_A_) of the pristine WO_2.72_ sensor is 0.23 eV, which is higher than that of the Cu SA/WO_2.72_ sensor (0.16 eV) (for a detailed explanation regarding the charge transport activation energy of sensor, see Supporting Information).

**Figure 5:**
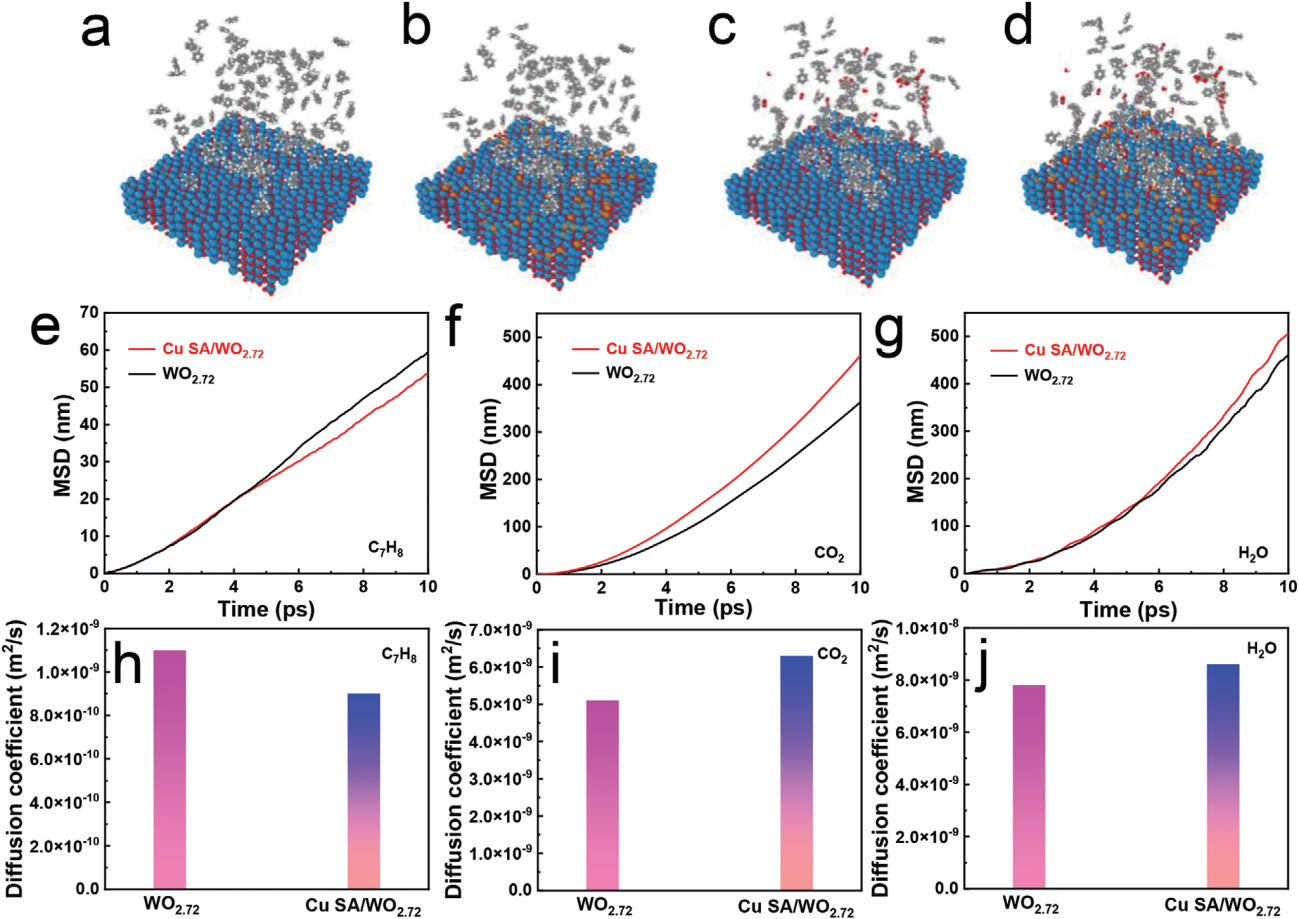
MD simulation snapshots for the dynamic process of toluene diffusion on a) pristine WO_2.72_ and b) Cu SA/WO_2.72_ samples. MD simulation snapshots for the dynamic process of carbon dioxide and water vapor diffusion on c) pristine WO_2.72_ and d) Cu SA/WO_2.72_ samples. Horizontal‐directional MSD of e) toluene, f) carbon dioxide, and g) water vapor on pristine WO_2.72_ and Cu SA/WO_2.72_ samples. Diffusion coefficient of h) toluene, i) carbon dioxide, and j) water vapor on pristine WO_2.72_ and Cu SA/WO_2.72_ samples.

To further understand the molecular‐level mechanism behind the improvement in toluene gas‐sensing characteristics, the molecular dynamic (MD) simulations are performed to study the dynamic process of toluene diffusion on pristine WO_2.72_ and Cu SA/WO_2.72_ samples (**Figure** **5**). The classical MD simulations are performed for 10 ps at 300 K to study how the introduction of Cu SAs as an active site affects the diffusion of toluene molecules in the horizontal‐directional.^[^ ^49^ ^]^ To quantitatively reflect the toluene molecules accumulation effect, the mean square displacement (MSD) is calculated to unveil the interaction between sensing material and toluene molecules. Interestingly, a clear difference in the horizontal‐directional diffusion behavior of toluene molecules, pristine WO_2.72_ shows a much higher MSD value than its corresponding Cu SA/WO_2.72_ sample, is observed (Figure 5e). Meanwhile, the diffusion coefficient of toluene over the pristine WO_2.72_ is greater than that of the Cu SA/WO_2.72_ sample (Figure 5h). Results reveal a stronger toluene affinity to Cu SACs‐containing sensing material systems. Consequently, a large number of toluene molecules accumulation around Cu SA/WO_2.72_ can greatly facilitate the toluene gas molecule's diffusion and chemisorption toward the active sites, which is particularly beneficial for the high response of the sensor. More remarkably, after 10 ps simulation, the corresponding MSD curves reveal much weaker carbon dioxide (or water vapor)–Cu SA/WO_2.72_ interaction relative to pristine WO_2.72_ (Figure 5f,g), suggesting the easy desorption of carbon dioxide (or water vapor) produced during the toluene gas detection process on Cu SA/WO_2.72_. The same phenomenon could be found for the diffusion coefficient (Figure 5i,j). As such, the carbon dioxide (or water vapor) can be easily desorbed from the Cu SA/WO_2.72_ sample surface,^[^ ^50^ ^]^ significant improvement for a quick release of the active sites can be expected from the sensitive layers, which has indeed been demonstrated by our Cu SA/WO_2.72_‐based sensors.

## 3 Conclusion

In summary, we have developed a novel, cost‐effective in situ‐annealing method for anchoring Cu SAs onto ultrathin WO_2.72_ nanowires. The resultant Cu SA/WO_2.72_‐based sensor exhibits remarkable response intensity (response of 1.9 at 10 ppb), high selectivity, and long‐term stability (negligible decay of response signal after 30 days of testing), which indicates that this sensing material is highly promising for practical applications in toluene gas sensing. Furthermore, our approach is supported by a combination of DFT calculations, MD simulations, and in situ IR spectroscopy, which provide valuable insights into the gas‐sensing mechanism of the Cu SA/WO_2.72_‐based sensor. Combined with in situ IR spectroscopy, DFT calculations demonstrate that the energy barrier of Cu SA/WO_2.72_ nanowires in the toluene oxidation reaction path is lower than that of pristine WO_2.72_. MD simulations reveal a stronger toluene affinity to Cu SACs‐containing sensing material systems. Overall, this study offers a promising strategy for designing and synthesizing highly sensitive and selective Cu SACs‐based gas‐sensing materials. Our approach provides a significant potential for the development of practical and high‐performance gas‐sensing and catalytic materials, suitable for large‐scale production.

## 4 Experimental Section

Experimental details are seen in the Supporting Information.

## Conflict of Interest

The authors declare no conflict of interest.

## Supporting information

Supporting Information

## Data Availability

The data that support the findings of this study are available from the corresponding author upon reasonable request.
