# Supplementary material for: Single‐Atom Cu Stabilized on Ultrathin WO2.72 Nanowire for Highly Selective and Ultrasensitive ppb‐Level Toluene Detection
Source: Adv Sci (Weinh). 2023 Jul 13;10(26):2302778. doi: 10.1002/advs.202302778 (PMC10502643; doi:10.1002/advs.202302778)
Supplement: Supplementary file 1 — Supporting Information [file ADVS-10-2302778-s001.pdf]

ADVANCED SCIENCE Open Access

# Supporting Information

for *Adv. Sci.*, DOI 10.1002/advs.202302778

Single-Atom Cu Stabilized on Ultrathin $WO_{2.72}$ Nanowire for Highly Selective and Ultrasensitive ppb-Level Toluene Detection

*Peng Wang, Shisong Guo, Zhixiang Hu, Licheng Zhou, Tiankun Li, Shiliang Pu*, Hui Mao, Hong Cai, Zhenfeng Zhu, Bingbing Chen, Hua-Yao Li* and Huan Liu*

# Supporting Information

**Single-atom Cu Stabilized on Ultrathin $WO_{2.72}$ Nanowire for Highly Selective and Ultrasensitive ppb-level Toluene Detection**

*Peng Wang, Shisong Guo, Zhixiang Hu, Licheng Zhou, Bingbing Chen, Tiankun Li, Shiliang Pu*, Hui Mao, Hong Cai, Zhenfeng Zhu, Hua-Yao Li*, and Huan Liu*

Dr. P. Wang, S. Guo, Dr. Z. Hu, Dr. L. Zhou, A. Prof. H. Li, and Prof. H. Liu

School of Integrated Circuits, Wuhan National Laboratory for Optoelectronics, Optics Valley Laboratory, Huazhong University of Science and Technology, 1037 Luoyu Road, Wuhan, Hubei 430074, P. R. China

E-mail: huayaoli@hust.edu.cn

T. Li, A. Prof. H. Li, and Prof. H. Liu

Wenzhou Key Laboratory of Optoelectronic Materials and Devices Application, Wenzhou Advanced Manufacturing Institute of HUST, 1085 Meiquan Road, Wenzhou, Zhejiang 325035, P. R. China

Dr. S. Pu, H. Mao, H. Cai, and Z. Zhu

Hikvision Research Institute, 555 Qianmo Road, Hangzhou, Zhejiang 310051, P. R. China

E-mail: pushiliang.hri@hikvision.com

Prof. B. Chen

School of Energy Science and Engineering, Nanjing Tech University, Nanjing, Jiangsu, 211816, P. R. China

**Experimental section**

**Chemicals.**

Tungsten hexachloride ($WCl_6$, 99%), cupric chloride ($CuCl_2$, 99.99%) and ethanol absolute ($CH_3CH_2OH$, 95%) were purchased from Shanghai Aladdin Biochemical Technology Co., Ltd.. Copper nanoparticles (Cu NPs, 99.9%) were purchased from Shanghai Maclin Biochemical Technology Co., Ltd.. The standard gases (toluene, paraxylene, formaldehyde, methane, carbon monoxide, carbon dioxide, ammonia, and synthetic air) were purchased from Wuhan Niuride Special Gas Co., Ltd.. Deionized water was prepared in the laboratory.

**Synthesis of ultrathin $WO_{2.72}$ nanowires.**

In a typical procedure, 0.25 g of $WCl_6$ was dissolved in 50 mL of absolute ethanol. After stirring for 30 min, it was transferred into a 100 mL of Teflon-lined autoclave and heated at 180°C for 24 h. Then, the product was washed alternately with deionized water and absolute ethanol. Finally, the precipitate is dried at 70°C for 8 h to obtain $WO_{2.72}$ sample.

**Synthesis of ultrathin Cu SA/$WO_{2.72}$ nanowires.**

0.25 g of $WCl_6$ and $CuCl_2$ (10 mg, 15 mg and 20 mg) were dissolved in 50 mL of absolute ethanol. After stirring for 30 min, it was transferred into a 100 mL of Teflon-lined autoclave and heated at 180°C for 24 h. Subsequently, the precipitate was washed alternately with deionized water and absolute ethanol, which was then dried at 70°C for 6 h. Finally, the annealing was conducted at 300°C for 1 h in air atmosphere. By adjusting the amount of $CuCl_2$, a series of samples with different Cu-loading amount were obtained and designated as Cu SA/$WO_{2.72}$-n, wherein n refers to the theoretical weight percentage of $CuCl_2$ to $WCl_6$ (10 mg, 15 mg and 20 mg of $CuCl_2$ for 4 wt.%, 6 wt.% and 8 wt.%, respectively). Based on inductively coupled plasma optical emission spectrometry (ICP-OES) testing, the Cu loads of Cu SA/$WO_{2.72}$-4%, Cu SA/$WO_{2.72}$-6% Cu SA/$WO_{2.72}$-8% samples were measured to be 0.765 wt.%, 1.108 wt.% and 1.375 wt.%, respectively.

**Synthesis of ultrathin Cu NPs/$WO_{2.72}$ nanowires.**

For comparison, 0.1 g of $WO_{2.72}$ powder was dispersed in 50 mL of deionized water at room temperature. After introducing 10 mg of Cu NPs, mixed treatment was carried out. Then, the precipitate was washed alternately with deionized water and absolute ethanol, and dried at 70°C for 8 h to obtain Cu NPs/$WO_{2.72}$ sample.

**Physicochemical characterization.**

X-ray diffraction (XRD) measurements were performed using powder X-ray diffractometer with Cu Kα radiation (Bruker D8 Advance). Fourier transform infrared (FTIR) spectra were carried out by Thermo Scientific Nicolet 6700 using KBr pellets. Raman spectra were measured by a Horiba LabRAM HR Evolution Raman system. X-ray photoelectron spectroscope (XPS) measurements were used by a Thermo Scientific K-Alpha with an Al source, and C 1s peak at 284.5 eV was used as reference. The ultraviolet-visible-near-infrared absorption spectrums were collected on a spectrophotometer (PE-Lamnda 35, Perkin EImer). The electron paramagnetic resonance (EPR) spectra were examined using a Bruker EMXplus-6/1spectrometer. To identify the Brunauer-Emmett-Teller (BET) surface area and pore size distribution, nitrogen adsorption-desorption isotherms (Micromeritics ASAP 2460) were collected. The morphology and energy-dispersive spectrometry (EDS) characterization were collected using transmission electron microscopy (JEOL JEM F200). The high-angle annular dark-field scanning transmission electron microscopy (HAADF-STEM) images and energy-dispersive X-ray spectroscopy (EDX) elemental mapping were recorded by using a FEI Themis Z. Elemental analysis of Cu in the solid sample was detected by an Agilent 5110 (OES) inductively coupled plasma optical emission spectrometry (ICP-OES). Thermal stability was recorded using a thermogravimetric differential thermal analyser (TG-DSC, STA 409 PC, Netzsch, Germany). Toluene adsorption-desorption isotherms were collected by a Quantachrome Autosorb-IQ-MP.

**XAFS measurement and data analysis.**

XAFS spectra at the Cu K-edge were measured at 1W1B station in Beijing Synchrotron Ration Facility (BSRF). The Cu K-edge XAFS data were recorded in a fluorescence mode. Cu foil, CuO, and $Cu_2O$ were used as references. All spectra were collected at room temperature. The acquired EXAFS data were processed according to the standard procedures using the ATHENA module implemented in the IFEFFIT software packages. The $k^3$-weighted EXAFS spectra were obtained by subtracting the post-edge background from the overall absorption and then normalizing with respect to the edge-jump step. Subsequently, $k^3$-weighted $\chi(k)$ data of Cu K-edge were Fourier transformed to real (R) space using a hanning windows (dk = 1.0 $Å^{-1}$) to separate the EXAFS contributions from different coordination shells. To obtain the quantitative structural parameters around central atoms, least-squares curve parameter fitting was performed using the ARTEMIS module of IFEFFIT software packages.

The following EXAFS equation was used:

$$\chi(k) = \sum_j \frac{N_j S_0^2 F_j(k)}{kR_j^2} \exp[-2k^2\sigma_j^2] \exp[\frac{-2R_j}{\lambda(k)}] \sin[2kR_j + \phi_j(k)]$$

where $S_0^2$ is the amplitude reduction factor, $F_j(k)$ is the effective curved-wave backscattering amplitude, $N_j$ is the number of neighbors in the $j^{th}$ atomic shell, $R_j$ is the distancebetween the X-ray absorbing central atom and the atoms in the $j^{th}$ atomic shell (backscatterer), $\lambda$ is the mean free path in Å, $\phi_j(k)$ is the phase shift (including the phase shift for each shell and the total central atom phase shift), $\sigma_j$ is the Debye-Waller parameter of the $j^{th}$ atomic shell (variation of distances around the average $R_j$). The functions $F_j(k)$, $\lambda$ and $\phi_j(k)$ were calculated with the ab initio code FEFF8.2.

**Gas sensing performance testing.**

The gas sensing properties were done in the laboratory. Figure S1 is a schematic diagram of the dynamic gas sensing performance testing system. The samples, prepared on alumina substrate (1 mm × 2 mm) with a printed gold electrode and a ruthenium oxide heater at the

backside (Figure S2), were put in a homemade chamber. All samples are drop-coated onto the alumina substrate with the gold electrode, until the gold electrode is completely covered by the sensing layer. The chamber is equipped with a gas inlet and an outlet, which are connected to a computer-controlled gas mixing system. The resistance of the sensor was continuously measured using a Keithley 2450 Source Meter with a computer interface. The sensor's operating temperature was determined by a voltage source, that is, the temperature of the heater can be altered by applying different voltages. The humidity was regulated by introducing dry synthetic air into a glass container filled with deionized water through bubbling. The relative humidity (RH) of the gas was regulated within a range of 0 to 70% by mixing the humid carrier gas with dry carrier gas at room temperature (25°C). For n-type semiconductors, the response was defined as Ra/Rg (Ra is the resistance of the sensor in air, and Rg is the resistance of the sensor in the target gas). The response and recovery time were defined as the time taken by the sensor to achieve 90% of the full magnitude change in resistance upon switching between target gas and air. [19]

**In situ infrared spectroscopy.**

The experimental setup of Diffuse Reflaxions Infrared Fourier Transformations Spectroscopy (DRIFTS) is combined with the dynamic gas sensing performance testing system to acquire the spectra continuously. The samples, prepared on alumina substrates, were put in a homemade chamber with a KBr window. The chamber was equipped with a gas inlet and an outlet connected to a computer-controlled gas mixing system. The DRIFTS spectra were acquired using a Vertex 80 v from Bruker with a nitrogen-cooled broad-band mercury cadmium telluride (MCT) detector with a spectral resolution of 4 $cm^{-1}$. The single-channel spectrum was continuously recorded every 10 min.

**Computational details.**

In this work, we had employed the first-principles to perform all density functional theory (DFT) calculations within the generalized gradient approximation (GGA) using the Perdew-

Burke-Ernzerhof (PBE) formulation. [1-3] We had chosen the projected augmented wave (PAW) potentials to describe the ionic cores and take valence electrons into account using a plane wave basis set with a kinetic energy cutoff of 400 eV. [4,5] Partial occupancies of the Kohn-Sham orbitals were allowed using the Gaussian smearing method and a width of 0.05 eV. The electronic energy was considered self-consistent when the energy change was smaller than $10^{-5}$ eV. A geometry optimization was considered convergent when the energy change was smaller than 0.05 eV $Å^{-1}$. In our structural model, U-correction was applied to the Cu and W atoms. The vacuum spacing in a direction perpendicular to the plane of the structure was 20 Å for the surfaces. The Brillouin zone integration was performed using 2×2×1 Monkhorst-Pack k-point sampling for a structure.

The adsorption energies ($\Delta E_{ads}$) would be defined as follows:

$$\Delta E_{ads} = E_{gas+surface} - (E_{surface} + E_{gas})$$

Where $E_{gas}$ is the energy of adsorbed gas molecules, $E_{surface}$ is the total energy of the material surface when the gas is not adsorbed, and $E_{gas+surface}$ is the total energy of the system after adsorbing the gas.

The free energy was calculated using the equation:

$$G = E_{ads} + ZPE - TS$$

where $G$, $E_{ads}$, ZPE and TS are the free energy, adsorption energy, zero-point energy and entropic contributions, respectively.

**MD simulations.**

In this work, two confined cases (pristine $WO_{2.72}$ and Cu SA/$WO_{2.72}$) were built for molecular dynamic (MD) simulations. Each confined case was constructed by using solid substrates and filled with 100 toluene. The substrates were set as rigid to ensure that the atoms of the substrates were fixed during the simulation. The temperature of each confined case was maintained at 300 K by using a Nosé-Hoover thermostat with a damping factor of 100 fs. The systems were

equilibrated for 10 ns to obtain a stable system. [6] After that, we added 20 molecules of carbon dioxide and 20 molecules of water to each system. And another 10 ns of simulation was performed for whole-case relaxation. The tip3p model was used for water. [7] The opls-aa force field was applied for toluene and carbon dioxide. [8] The Interface Force Field was used for solid substrates. The Lorentz-Berthelot mixing rule was adopted for the van der Waals interactions of different kinds of atoms. All the cases were placed in periodic orthogonal boxes. And all the MD simulations were performed by using LAMMPS software package. [9]

**Figure S1.** Schematic diagram of the dynamic gas sensing performance testing system. (The mass flow controller is denoted by MFC)

**Figure S2.** Structure diagram of the sensor plane electrode: (a) gold electrode and (b) ruthenium oxide heater at the backside.

**Figure S3.** Characterization of pristine $WO_{2.72}$. (a,b) TEM image, (c) HRTEM image and (d-g) EDS maps.

**Figure S4.** The local magnification TEM image of Cu SA/$WO_{2.72}$-4%.

**Figure S5.** $N_2$ adsorption-desorption isotherms of pristine $WO_{2.72}$, Cu SA/$WO_{2.72}$-4%, Cu SA/$WO_{2.72}$-6% and Cu SA/$WO_{2.72}$-8% samples.

**Figure S6.** Pore size distribution of pristine $WO_{2.72}$, Cu SA/$WO_{2.72}$-4%, Cu SA/$WO_{2.72}$-6% and Cu SA/$WO_{2.72}$-8% samples.

**Figure S7.** Characterization of Cu SA/$WO_{2.72}$-4%. (a,b) EDS spectrum and (c-f) EDS maps.

**Figure S8.** TEM image of reference sample Cu NPs/$WO_{2.72}$.

**Figure S9.** Characterization of reference sample Cu SA/$WO_{2.72}$-6%. (a) AC HAADF-STEM image and (b-e) corresponding EDX elemental mapping for elements W, Cu and O.

**Figure S10.** Characterization of reference sample Cu SA/$WO_{2.72}$-8%. (a) AC HAADF-STEM image and (b-e) corresponding EDX elemental mapping for elements W, Cu and O.

**Figure S11.** XRD patterns of pristine $WO_{2.72}$, Cu SA/$WO_{2.72}$-4%, Cu SA/$WO_{2.72}$-6%, Cu SA/$WO_{2.72}$-8% and Cu NPs/$WO_{2.72}$ samples.

**Figure S12.** Raman spectra of pristine $WO_{2.72}$, Cu SA/$WO_{2.72}$-4%, Cu SA/$WO_{2.72}$-6% and Cu SA/$WO_{2.72}$-8% samples.

**Figure S13.** EPR spectra of pristine $WO_{2.72}$, Cu SA/$WO_{2.72}$-4%, Cu SA/$WO_{2.72}$-6% and Cu SA/$WO_{2.72}$-8% samples.

**Figure S14**. Ultraviolet-visible-near-infrared absorption spectra of pristine $WO_{2.72}$, Cu SA/$WO_{2.72}$-4%, Cu SA/$WO_{2.72}$-6% and Cu SA/$WO_{2.72}$-8% samples.

**Figure S15.** FTIR spectra of pristine $WO_{2.72}$, Cu SA/$WO_{2.72}$-4%, Cu SA/$WO_{2.72}$-6% and Cu SA/$WO_{2.72}$-8% samples.

**Figure S16.** The high-resolution XPS spectra of (a) W 4f peaks, (b) O 1s peaks and (c) Cu 2p peaks for pristine $WO_{2.72}$, Cu SA/$WO_{2.72}$-6% and Cu SA/$WO_{2.72}$-8% samples.

**Figure S17.** Fourier transformed EXAFS spectra of the Cu K edge for Cu foil, CuO, $Cu_2O$ and Cu SA/$WO_{2.72}$-4% in K space.

**Figure S18.** WT-EXAFS plot of $Cu_2O$.

**Figure S19.** Thermogravimetric-differential scanning calorimetry (TG-DSC) curves of (a) pristine $WO_{2.72}$ and (b) Cu SA/$WO_{2.72}$.

**Figure S20.** Operation temperature optimization for pristine $WO_{2.72}$, Cu SA/$WO_{2.72}$-4%, Cu SA/$WO_{2.72}$-6%, Cu SA/$WO_{2.72}$-8% and Cu NPs/$WO_{2.72}$ sensors.

**Figure S21.** Dynamic response curves of pristine $WO_{2.72}$, Cu SA/$WO_{2.72}$-4%, Cu SA/$WO_{2.72}$-6%, Cu SA/$WO_{2.72}$-8% and Cu NPs/$WO_{2.72}$ sensors to 2.5 ppm toluene at 160°C.

**Figure S22.** (a) Response time and (b) recovery time of pristine $WO_{2.72}$, Cu SA/$WO_{2.72}$-4% and Cu NPs/$WO_{2.72}$ sensors to 2.5 ppm toluene. (c) Response of pristine $WO_{2.72}$, Cu SA/$WO_{2.72}$-4% and Cu NPs/$WO_{2.72}$ sensors to 2.5 ppm toluene.

**Figure S23.** The response of pristine $WO_{2.72}$, Cu SA/$WO_{2.72}$-4% and Cu NPs/$WO_{2.72}$ sensors to toluene in different concentrations (250-10000 ppb).

**Figure S24.** (a) Dynamic resistance curves of pristine $WO_{2.72}$, Cu SA/$WO_{2.72}$-4% and Cu NPs/$WO_{2.72}$ sensors toward toluene at different concentrations (25-400 ppm). (b) The relative response versus the toluene concentration illustration of the pristine $WO_{2.72}$, Cu SA/$WO_{2.72}$-4% and Cu NPs/$WO_{2.72}$ sensors.

**Figure S25.** Response of Cu SA/$WO_{2.72}$-4% sensor to lower concentration toluene (10, 25, 50 and 100 ppb).

**Figure S26.** Calculation of the limit of detection (LOD). The linear fitting of pristine $WO_{2.72}$ sensor response with toluene concentrations in the linear region, the slope is 0.0008 $ppb^{-1}$.

**Figure S27.** Calculation of the limit of detection (LOD). The linear fitting of Cu NPs/$WO_{2.72}$ sensor response with toluene concentrations in the linear region, the slope is 0.0011 $ppb^{-1}$.

**Figure S28.** Calculation of the limit of detection (LOD). The linear fitting of Cu SA/$WO_{2.72}$-4% sensor response with toluene concentrations in the linear region, the slope is 0.0018 $ppb^{-1}$.

**Figure S29.** Repeatability tests of pristine $WO_{2.72}$, Cu SA/$WO_{2.72}$-4% and Cu NPs/$WO_{2.72}$ sensors to 250 ppm toluene.

**Figure S30.** Characterization of sensitive material after the continuous gas sensing test to 2.5 ppm toluene gas for thirty days based on the Cu SA/$WO_{2.72}$-4% sensor. (a) TEM image, (b) AC HAADF-STEM image, (c) AC HAADF-STEM image and (d-g) corresponding EDX elemental mapping for elements W, Cu and O.

**Figure S31.** Real-time sensing curves of (a) pristine $WO_{2.72}$, (b) Cu NPs/$WO_{2.72}$, (c) Cu SA/$WO_{2.72}$-4% sensors to 2.5 ppm toluene at different relative humidity (RH), and (d) the relationship between response and RH.

**Figure S32.** The crystal structure models are established for (a) pristine $WO_{2.72}$, (b) Cu SA/$WO_{2.72}$ and (c) Cu NPs/$WO_{2.72}$.

**Figure S33.** The adsorption energy ($E_{ads}$) of Cu site, W site, oxygen vacancy ($V_o$) site and O site toward toluene gas molecule on Cu SA/$WO_{2.72}$ crystal structure.

**Figure S34.** The adsorption energies ($E_{ads}$) of Cu SA/$WO_{2.72}$ crystal structure toward different gas molecules (T is toluene, P is paraxylene, F is formaldehyde, M is methane, CM is carbon monoxide, CD is carbon dioxide and A is ammonia).

**Figure S35.** The adsorption energies ($E_{ads}$) of Cu NPs/$WO_{2.72}$ crystal structure toward different gas molecules (T is toluene, P is paraxylene, F is formaldehyde, M is methane, CM is carbon monoxide, CD is carbon dioxide and A is ammonia).

**Figure S36.** The adsorption energies ($E_{ads}$) of pristine $WO_{2.72}$ crystal structure toward different gas molecules (T is toluene, P is paraxylene, F is formaldehyde, M is methane, CM is carbon monoxide, CD is carbon dioxide and A is ammonia).

**Figure S37.** The adsorption energies ($E_{ads}$) of toluene on the pristine $WO_{2.72}$ and Cu SA/$WO_{2.72}$.

**Figure S38.** Comparative analysis of toluene adsorption-desorption isotherms to pristine $WO_{2.72}$ and Cu SA/$WO_{2.72}$.

**Figure S39.** Fitting of Langmuir and Freundlich isothermal model to pristine $WO_{2.72}$ sensor response.

**Figure S40.** Fitting of Langmuir and Freundlich isothermal model to Cu SA/$WO_{2.72}$ sensor response.

**Figure S41.** Proposed toluene sensing and conversion mechanism on Cu SA/$WO_{2.72}$-based sensor.

**Figure S42.** In situ DRIFTS spectra (2000-4000 $cm^{-1}$) for the oxidation of toluene over (a) pristine $WO_{2.72}$ and (b) Cu SA/$WO_{2.72}$ samples at different reaction time.

**Figure S43.** In situ DRIFTS spectra for the oxidation of toluene over pristine $WO_{2.72}$ and Cu SA/$WO_{2.72}$ samples at 80 mins.

**Figure S44.** Fitting of thermodynamic equation to pristine $WO_{2.72}$ sensor response.

**Figure S45.** Fitting of thermodynamic equation to Cu SA/$WO_{2.72}$ sensor response.

**Figure S46.** Arrhenius plot of log resistance versus reciprocal temperature for pristine $WO_{2.72}$ sensor.

**Figure S47.** Arrhenius plot of log resistance versus reciprocal temperature for Cu SA/$WO_{2.72}$ sensor.

**Figure S48.** MD simulation snapshot for the dynamic process of toluene diffusion on pristine $WO_{2.72}$ sample.

**Figure S49.** MD simulation snapshot for the dynamic process of carbon dioxide and water vapor diffusion on pristine $WO_{2.72}$ sample.

**Figure S50.** MD simulation snapshot for the dynamic process of toluene diffusion on Cu SA/$WO_{2.72}$ sample.

**Figure S51.** MD simulation snapshot for the dynamic process of carbon dioxide and water vapor diffusion on Cu SA/$WO_{2.72}$ sample.

**Table S1.** Textual properties of pristine $WO_{2.72}$, Cu SA/$WO_{2.72}$-4%, Cu SA/$WO_{2.72}$-6% and Cu SA/$WO_{2.72}$-8% samples.

**Table S2.** EXAFS fitting parameters at the Cu K-edge for Cu SA/$WO_{2.72}$-4% ($S_0^2$=0.91).

**Table S3.** The peak area ratio of $O_{latt}$, $O_{sur}$ and $O_{ads}$ for pristine $WO_{2.72}$, Cu SA/$WO_{2.72}$-4%, Cu SA/$WO_{2.72}$-6% and Cu SA/$WO_{2.72}$-8% samples.

**Table S4.** The peak area ratio of $W^{5+}$ and $W^{6+}$ for pristine $WO_{2.72}$, Cu SA/$WO_{2.72}$-4%, Cu SA/$WO_{2.72}$-6% and Cu SA/$WO_{2.72}$-8% samples.

**Table S5.** Properties comparison of toluene sensors.

**Table S6.** Infrared vibration information of toluene related to intermediate species for the oxidation over pristine $WO_{2.72}$ and Cu SA/$WO_{2.72}$ samples. [48-50]

**Table S7.** Langmuir and Freundlich response model parameters of sensor.

**Table S8.** Thermodynamic parameters of sensor.

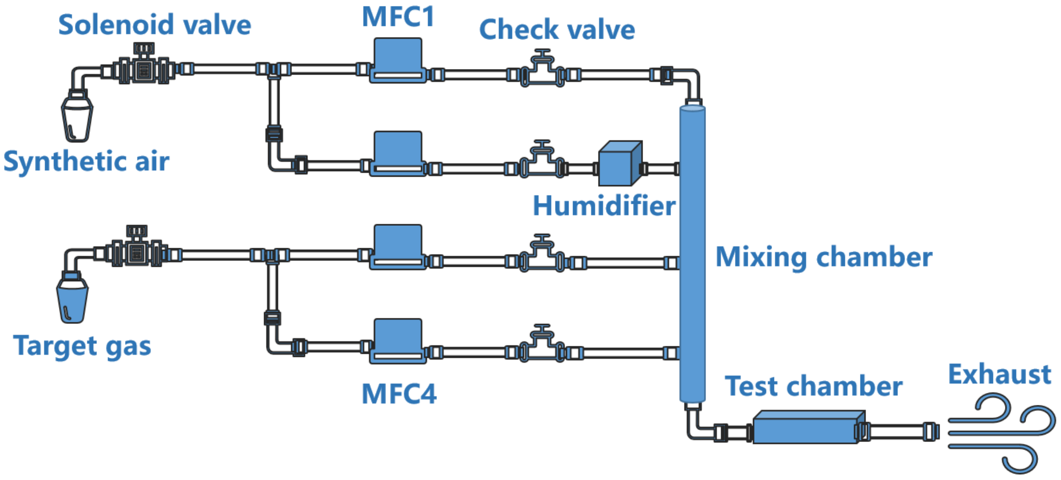


**Figure S1.** Schematic diagram of the dynamic gas sensing performance testing system. (The mass flow controller is denoted by MFC)

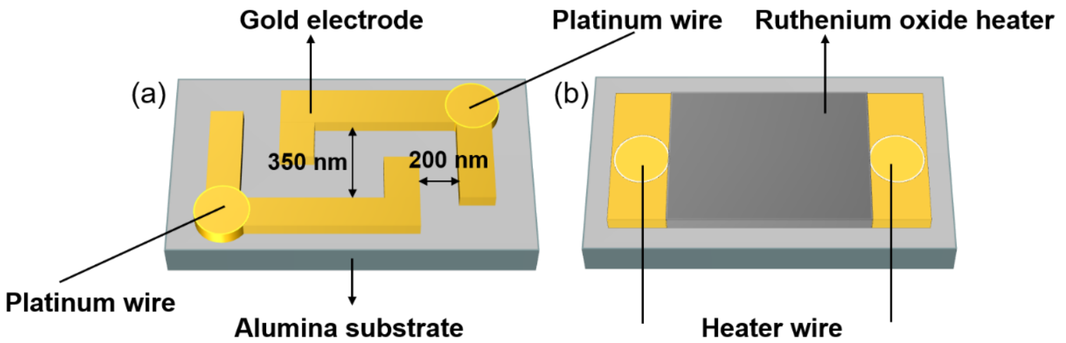


**Figure S2**. Structure diagram of the sensor plane electrode: (a) gold electrode and (b) ruthenium oxide heater at the backside.

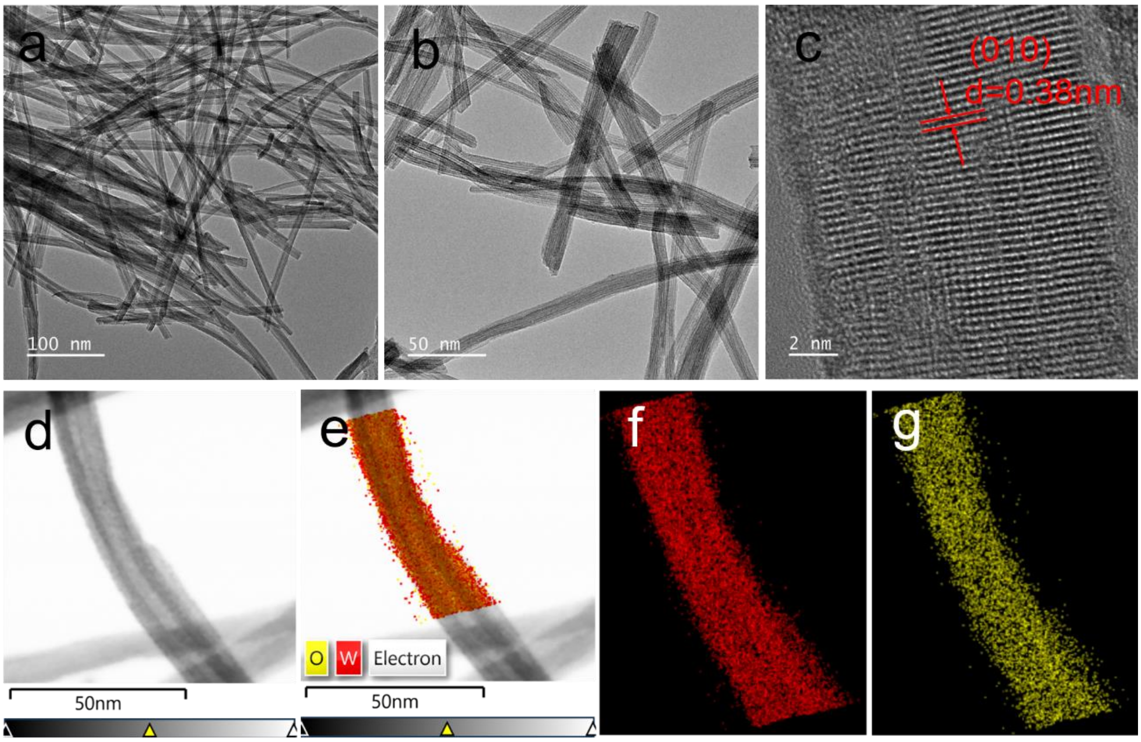


**Figure S3.** Characterization of pristine $WO_{2.72}$. (a,b) TEM image, (c) HRTEM image and (d-g) EDS maps.

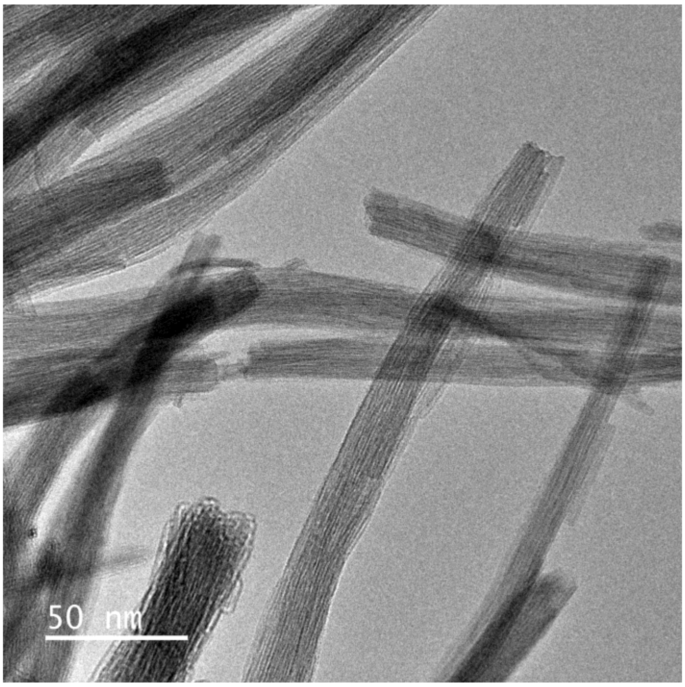


**Figure S4.** The local magnification TEM image of Cu SA/$WO_{2.72}$-4%.

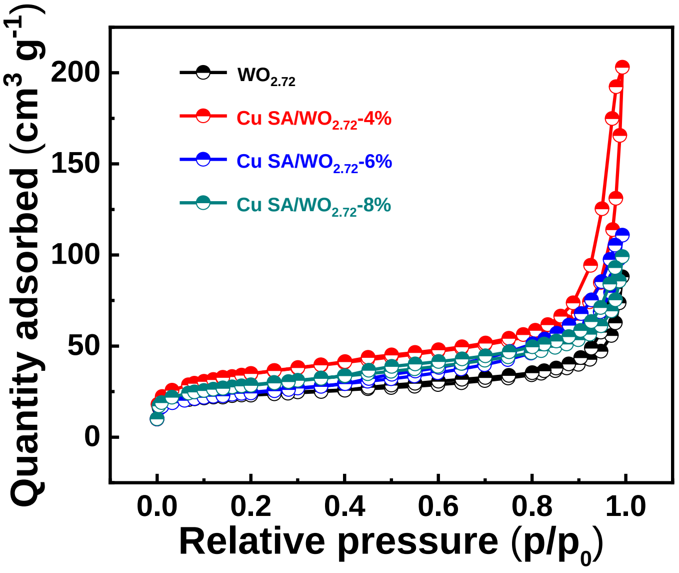


**Figure S5.** $N_2$ adsorption-desorption isotherms of pristine $WO_{2.72}$, Cu SA/$WO_{2.72}$-4%, Cu SA/$WO_{2.72}$-6% and Cu SA/$WO_{2.72}$-8% samples.

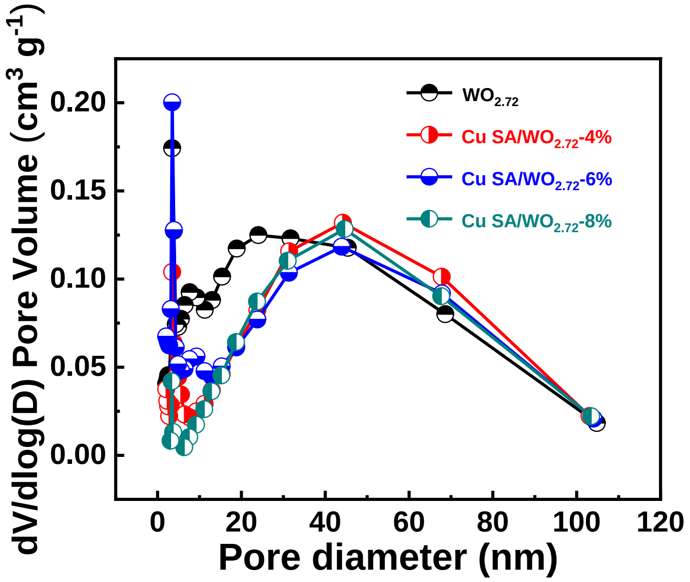


**Figure S6.** Pore size distribution of pristine $WO_{2.72}$, Cu SA/$WO_{2.72}$-4%, Cu SA/$WO_{2.72}$-6% and Cu SA/$WO_{2.72}$-8% samples.

All as-synthesized samples display type IV isotherms with H3 hysteresis loops at relatively high pressure, indicating the presence of mesoporous in the nanowires. [10] As is evident from the Brunauer-Emmett-Teller (BET) analysis, the Cu SA/$WO_{2.72}$-4% has the largest surface area with a value of 98.72 $m^2 \cdot g^{-1}$, compared to 78.57, 89.48 and 85.00 $m^2 \cdot g^{-1}$ of pristine $WO_{2.72}$, Cu

SA/$WO_{2.72}$-6% and Cu SA/$WO_{2.72}$-8% samples, respectively. Additionally, the pore size distribution changes slightly after Cu SAs introduction, the Cu SA/$WO_{2.72}$-4%, Cu SA/$WO_{2.72}$-6% and Cu SA/$WO_{2.72}$-8% samples have uniform pore diameter centered about 44.6 nm, demonstrating its characteristic mesoporous structure. Obviously, high surface area, porous structure, and evenly distributed active sites are beneficial for improving gas sensing performance.

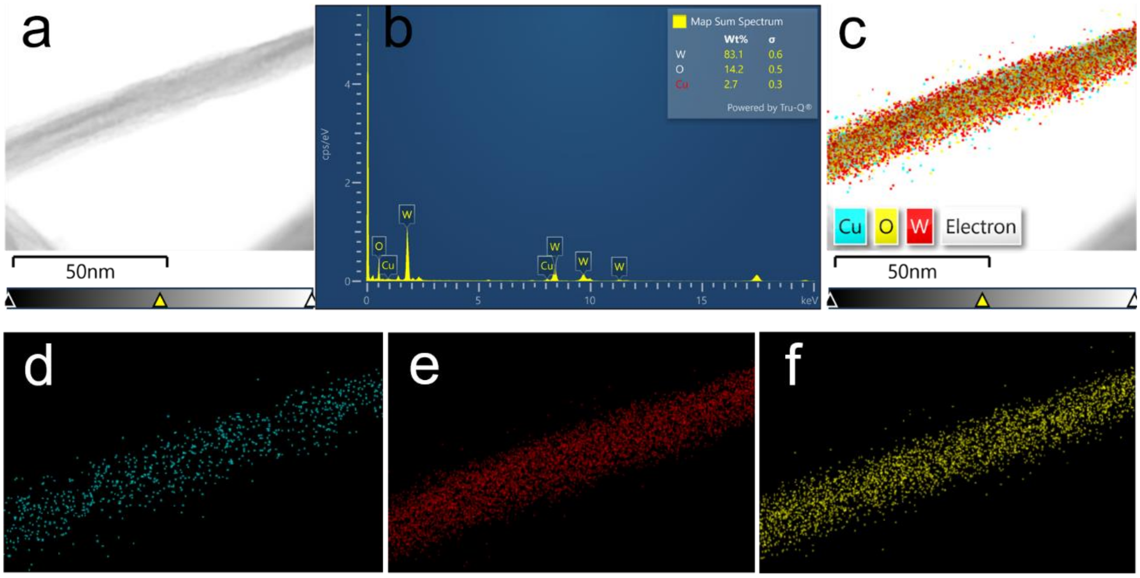


**Figure S7.** Characterization of Cu SA/$WO_{2.72}$-4%. (a,b) EDS spectrum and (c-f) EDS maps.

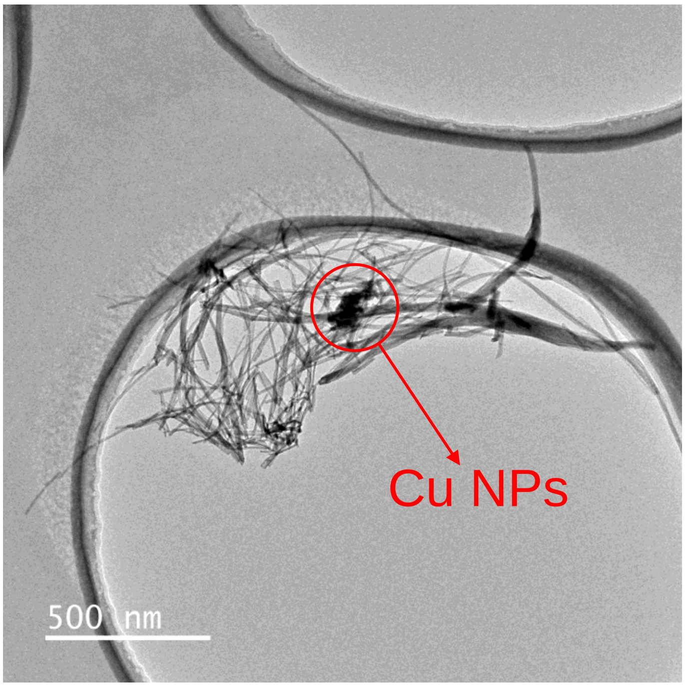


**Figure S8.** TEM image of reference sample Cu NPs/$WO_{2.72}$.

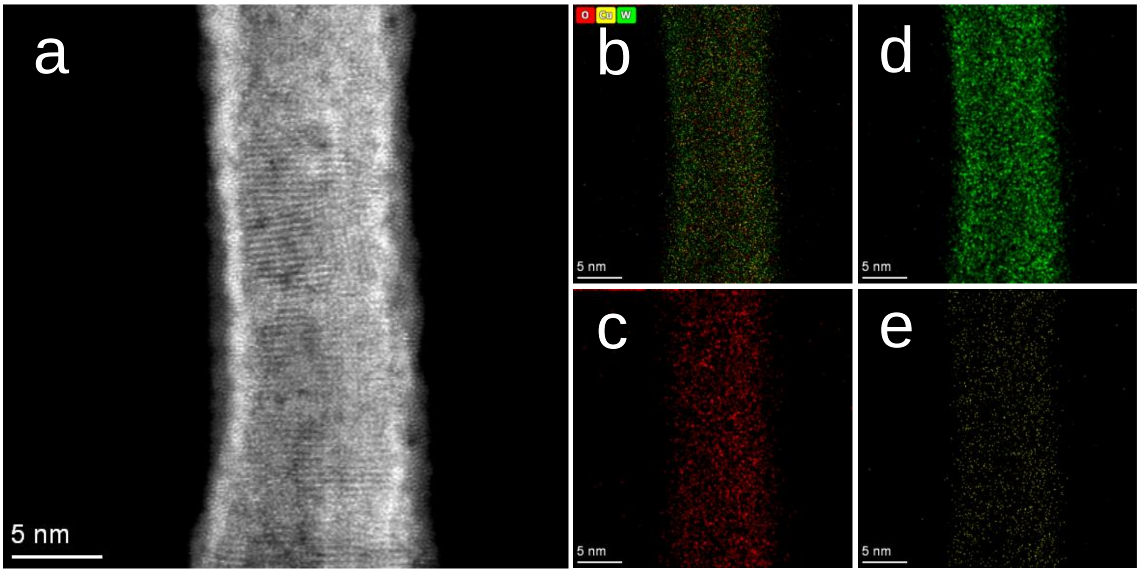


**Figure S9.** Characterization of reference sample Cu SA/$WO_{2.72}$-6%. (a) AC HAADF-STEM image and (b-e) corresponding EDX elemental mapping for elements W, Cu and O.

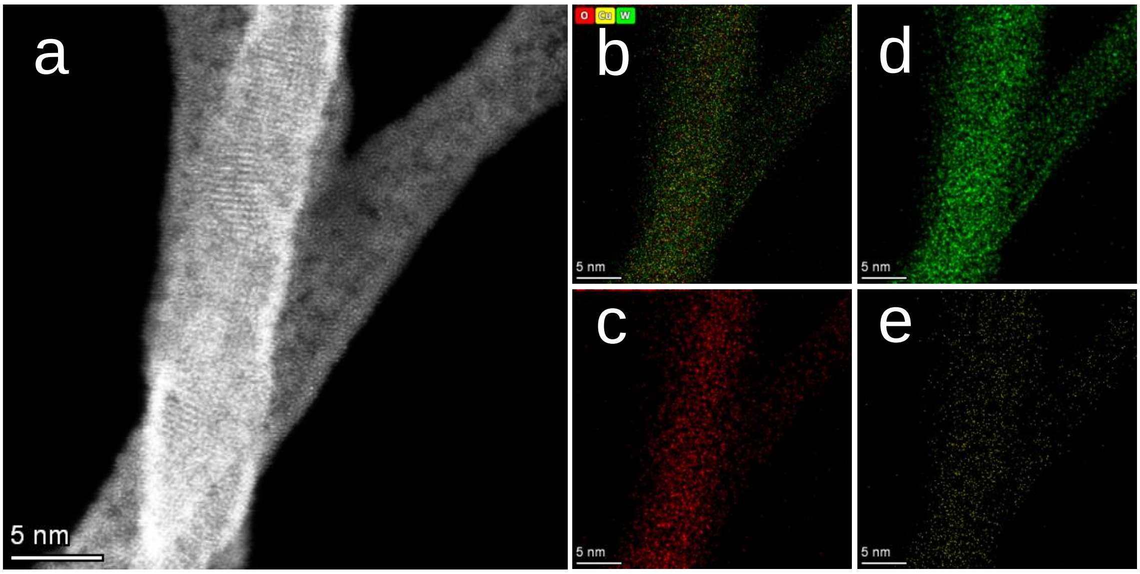


**Figure S10.** Characterization of reference sample Cu SA/$WO_{2.72}$-8%. (a) AC HAADF-STEM image and (b-e) corresponding EDX elemental mapping for elements W, Cu and O.

The AC HAADF-STEM images show that reference samples Cu SA/$WO_{2.72}$-6% and Cu SA/$WO_{2.72}$-8% are one-dimensional nanowire structure. Meanwhile, no Cu nanoparticles or clusters could be found. Moreover, the EDX elemental mapping results reveal that the main elements of reference samples (Cu SA/$WO_{2.72}$-6% and Cu SA/$WO_{2.72}$-8%) are W, Cu and O. And it can be distinctly observed that Cu atoms are homogeneously distributed over as-synthesized nanowires.

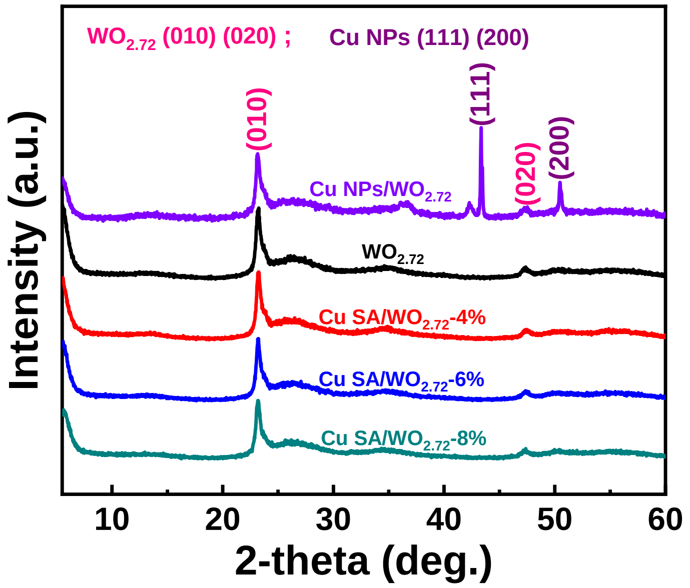


**Figure S11.** XRD patterns of pristine $WO_{2.72}$, Cu SA/$WO_{2.72}$-4%, Cu SA/$WO_{2.72}$-6%, Cu SA/$WO_{2.72}$-8% and Cu NPs/$WO_{2.72}$ samples.

For XRD patterns, the appearance of $WO_{2.72}$ is supported by the visible diffraction peaks from (010) and (020) planes. Meanwhile, the XRD patterns agree with monoclinic $WO_{2.72}$ phase (space group P2m, a = 18.318, b = 3.784, and c = 14.028 Å). [11] With the increasing Cu SA loadings, no apparent peaks of Cu NPs or other phases could be detected. However, for Cu NPs/$WO_{2.72}$, the typical diffraction peaks of Cu NPs, corresponding to the (111) and (200) planes, are observed. [12] In addition, XRD analysis results reveal that introduction of Cu SAs does not alter the crystal structure of pristine $WO_{2.72}$. Notably, the existence of Cu SAs has a certain effect on the crystallinity.

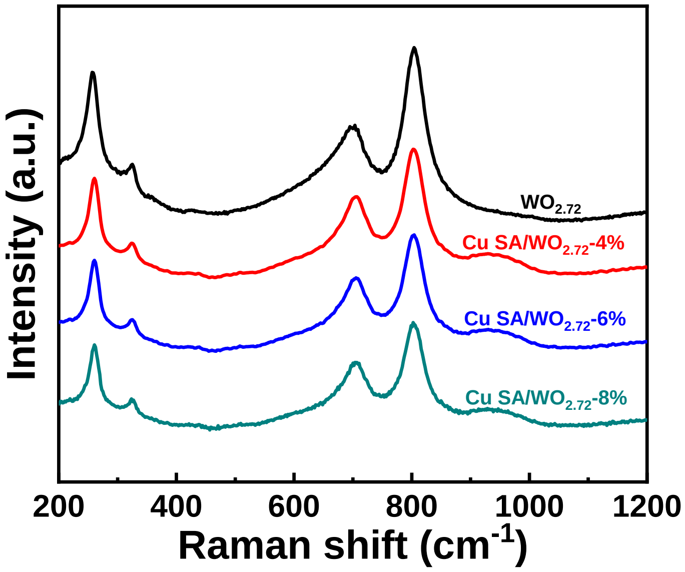


**Figure S12.** Raman spectra of pristine $WO_{2.72}$, Cu SA/$WO_{2.72}$-4%, Cu SA/$WO_{2.72}$-6% and Cu SA/$WO_{2.72}$-8% samples.

Raman spectra suggest that the Cu SAs have a negligible effect on the monoclinic crystal structure. The bands around 256 and 325 $cm^{-1}$ are ascribed to the O-W-O bending mode, and the bands at about 703 and 804 $cm^{-1}$ are attributable to the W-O stretching mode. [13,14] We could see that, with the increasing Cu SA loadings, the peak intensity of the as-synthesized samples gradually decreases, which is deterioration of the crystallinity. It is worth noting that, compared with pristine $WO_{2.72}$, the positions of the peaks around 256, 325 and 703 $cm^{-1}$ shift to a high wavenumber after being loaded with Cu SAs, representing a blue-shift. [15] This phenomenon indicates that the introduction of Cu SAs increases the number of oxygen vacancies, i.e. the local atomic structure disorders/defects. [16-18]

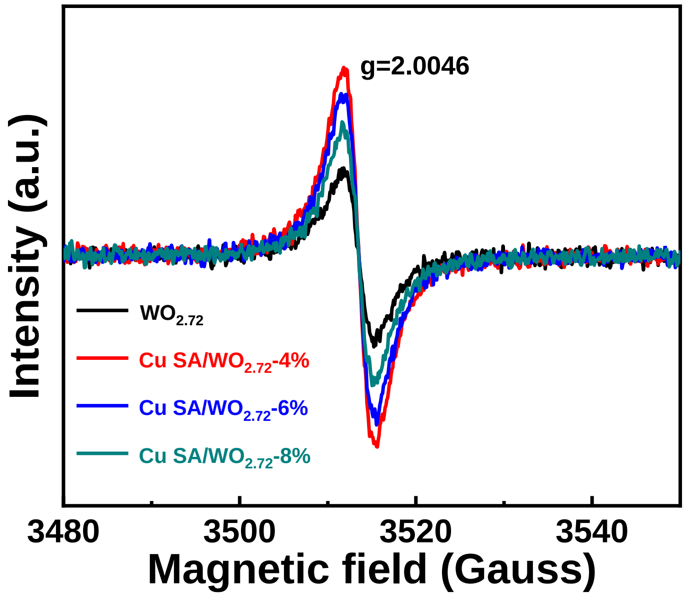


**Figure S13.** EPR spectra of pristine $WO_{2.72}$, Cu SA/$WO_{2.72}$-4%, Cu SA/$WO_{2.72}$-6% and Cu SA/$WO_{2.72}$-8% samples.

An apparent EPR signal at a g factor of 2.0046 is observed, demonstrating the unpaired electrons in the oxygen vacancy sites. [19] Compared with pristine $WO_{2.72}$, the EPR signals are stronger after Cu SAs introduction, implying more oxygen vacancies. The more oxygen vacancies could promote toluene gas adsorption, which is conducive to the enhancement of gas sensing performance.

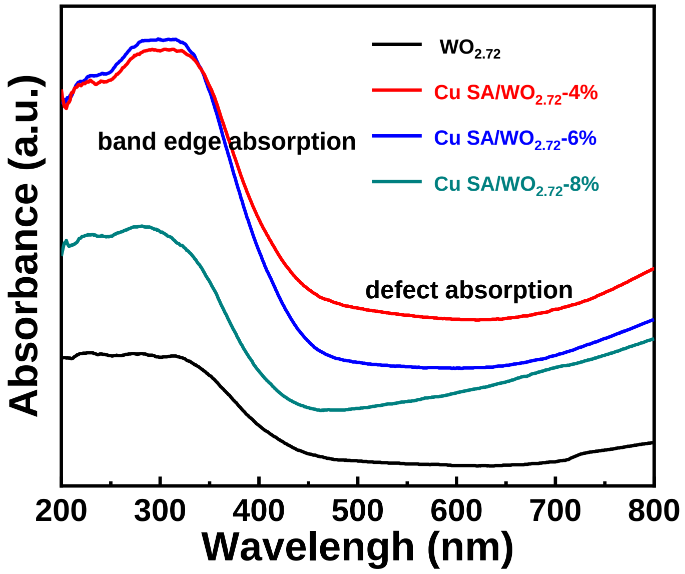


**Figure S14**. Ultraviolet-visible-near-infrared absorption spectra of pristine $WO_{2.72}$, Cu SA/$WO_{2.72}$-4%, Cu SA/$WO_{2.72}$-6% and Cu SA/$WO_{2.72}$-8% samples.

The higher broad absorption tails are appeared for Cu SA/$WO_{2.72}$-4%, Cu SA/$WO_{2.72}$-6% and Cu SA/$WO_{2.72}$-8% samples in the range of 400~800 nm, compared with pristine $WO_{2.72}$. These results indicate the oxygen vacancies increase after Cu SAs introduction.

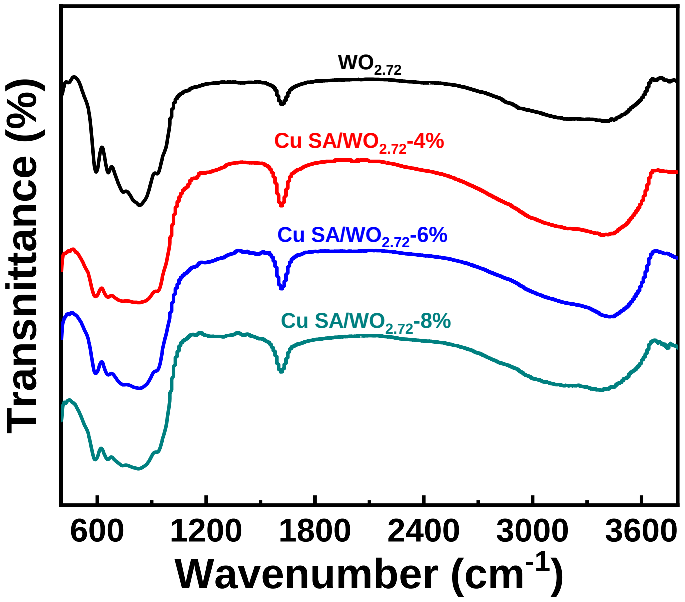


**Figure S15.** FTIR spectra of pristine $WO_{2.72}$, Cu SA/$WO_{2.72}$-4%, Cu SA/$WO_{2.72}$-6% and Cu SA/$WO_{2.72}$-8% samples.

For FTIR spectra, the bands match well with the bending vibration of peroxy W-O-O-W groups (592 $cm^{-1}$) and the stretching vibration of W-O-W groups (665 $cm^{-1}$), as well as the bending vibration of O-W-O groups (823 $cm^{-1}$), in agreement with the typical vibrations of $WO_{2.72}$ previously reported. [20] In addition, the bands (1630 and 3410 $cm^{-1}$) correspond to the bending vibration of W-OH groups from adsorbed water molecules and -OH groups in absorbed water molecules, respectively. [21] Compared with pristine $WO_{2.72}$, the peak shapes of Cu SA/$WO_{2.72}$-4%, Cu SA/$WO_{2.72}$-6% and Cu SA/$WO_{2.72}$-8% samples at about 823 $cm^{-1}$ become wider, which may be due to the decrease in crystallinity. [22]

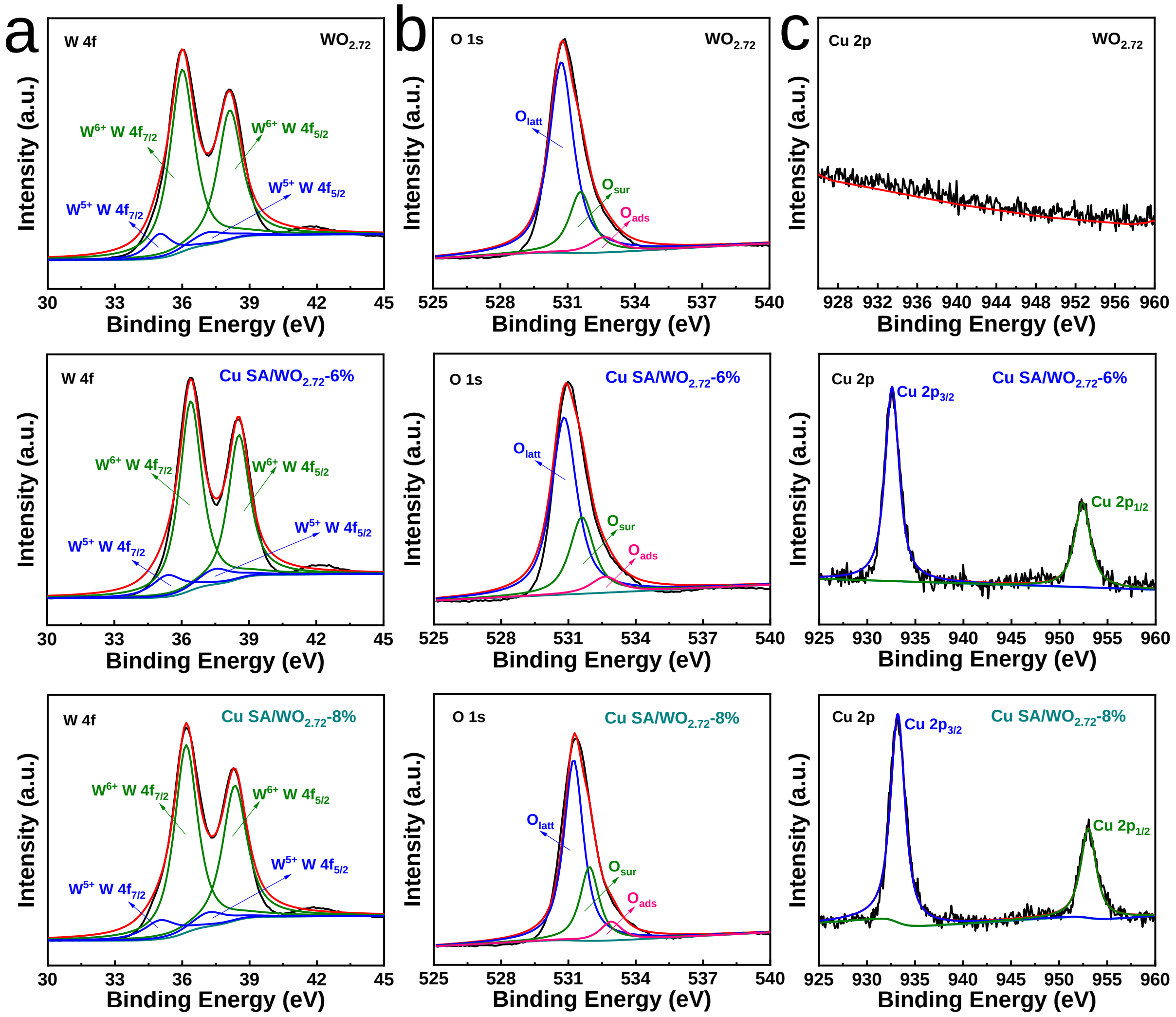


**Figure S16.** The high-resolution XPS spectra of (a) W 4f peaks, (b) O 1s peaks and (c) Cu 2p peaks for pristine $WO_{2.72}$, Cu SA/$WO_{2.72}$-6% and Cu SA/$WO_{2.72}$-8% samples.

The appearance of $W^{5+}$ peaks is ascribed to the unsaturated W-O bond in $WO_{2.72}$ crystalline structure. The larger the peak area of $W^{5+}$, the more content of defective oxygen. The Cu SAs anchored on the ultrathin $WO_{2.72}$ nanowires increase oxygen vacancy.

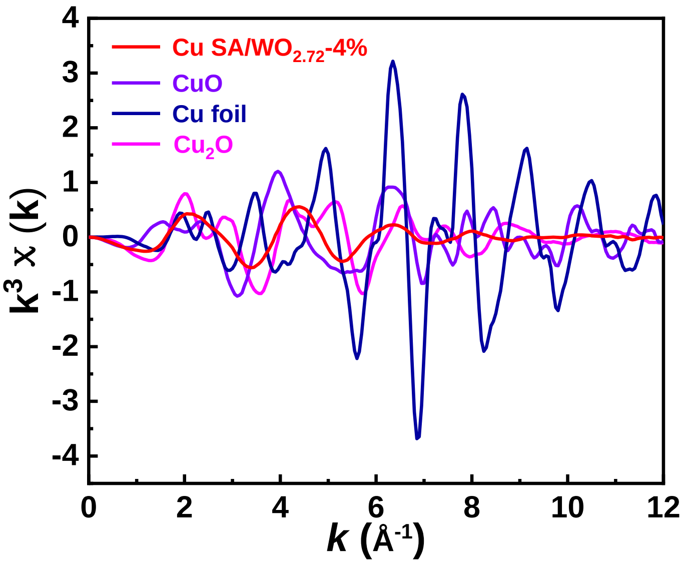


**Figure S17.** Fourier transformed EXAFS spectra of the Cu K edge for Cu foil, CuO, $Cu_2O$ and Cu SA/$WO_{2.72}$-4% in K space.

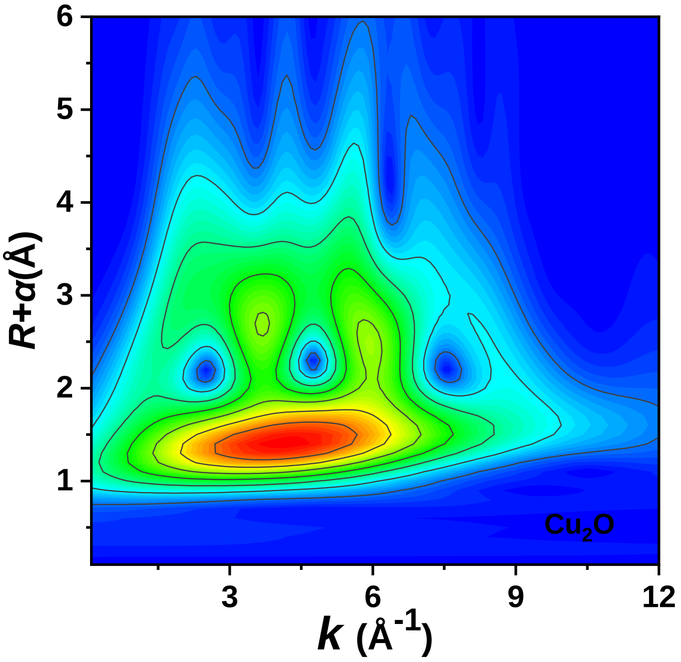


**Figure S18.** WT-EXAFS plot of $Cu_2O$.

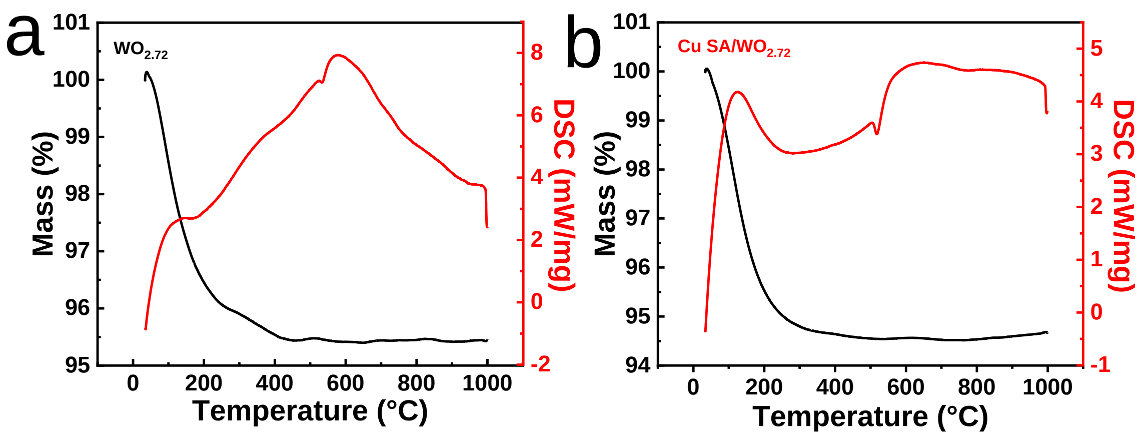


**Figure S19.** Thermogravimetric-differential scanning calorimetry (TG-DSC) curves of (a) pristine $WO_{2.72}$ and (b) Cu SA/$WO_{2.72}$.

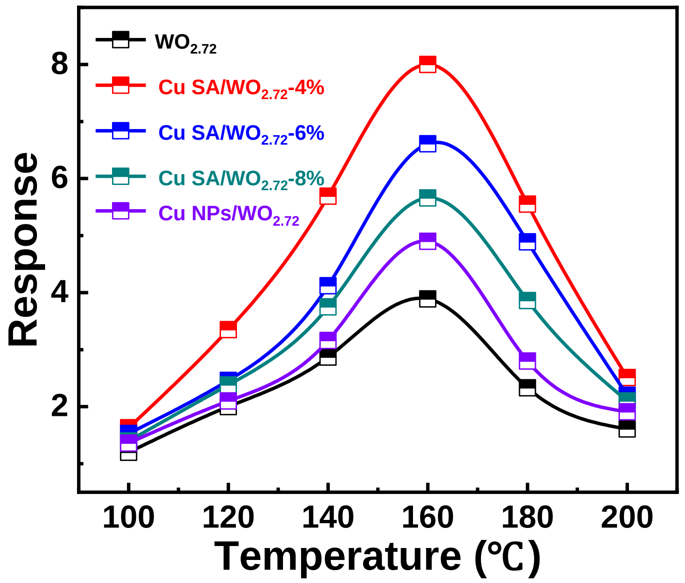


**Figure S20.** Operation temperature optimization for pristine $WO_{2.72}$, Cu SA/$WO_{2.72}$-4%, Cu SA/$WO_{2.72}$-6%, Cu SA/$WO_{2.72}$-8% and Cu NPs/$WO_{2.72}$ sensors.

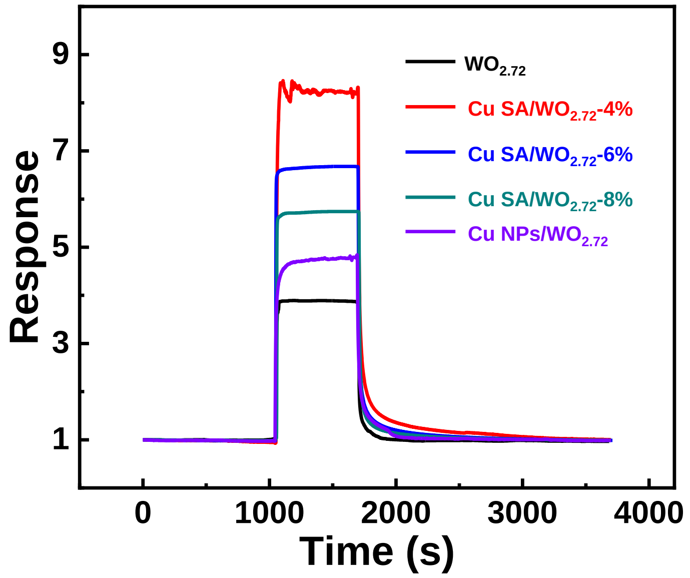

**Figure S21.** Dynamic response curves of pristine $WO_{2.72}$, Cu SA/$WO_{2.72}$-4%, Cu SA/$WO_{2.72}$-6%, Cu SA/$WO_{2.72}$-8% and Cu NPs/$WO_{2.72}$ sensors to 2.5 ppm toluene at 160°C.

According to the results of thermal stability analysis (Figure S19), the gas sensing properties of sensors operating at different temperatures, ranging from 100 to 200°C, are evaluated. Figure S20 shows temperature-dependent responses of pristine $WO_{2.72}$, Cu SA/$WO_{2.72}$-4%, Cu SA/$WO_{2.72}$-6%, Cu SA/$WO_{2.72}$-8% and Cu NPs/$WO_{2.72}$ sensors to 2.5 ppm toluene. We could observe that, the response values first increase and then decrease with increasing temperature, showing a “volcano” shape. The results show that optimum operating temperature is 160°C. At the optimum operating temperature conditions, the dynamic response curves of the pristine $WO_{2.72}$, Cu SA/$WO_{2.72}$-4%, Cu SA/$WO_{2.72}$-6%, Cu SA/$WO_{2.72}$-8% and Cu NPs/$WO_{2.72}$ sensors toward 2.5 ppm toluene are compared (Figure S21). As a result, the introduction of different types Cu species (Cu SAs and Cu NPs) increases the response values, which is a clear demonstration that the catalytic action of Cu species improves the toluene gas sensing performance. More remarkably, the response values of the Cu SA/$WO_{2.72}$-4%, Cu SA/$WO_{2.72}$-6% and Cu SA/$WO_{2.72}$-8% sensors are all greater than those of Cu NPs/$WO_{2.72}$ sensor, which shows the easy aggregation of Cu NPs reduces active sites exposed for toluene gas sensing reaction. However, with the increase of Cu SA loadings, the response values first increase and then decrease, which could be attributed to that excessive Cu SAs reduce the surface area of sensitive materials overall, therefore reduction in the reaction site results in lessened resistance change. Note that the Cu SA/$WO_{2.72}$-4% sensor has the largest response value. Next, the gas sensing performance evaluation of the sensors in the manuscript is performed at an optimum operating temperature of 160°C.

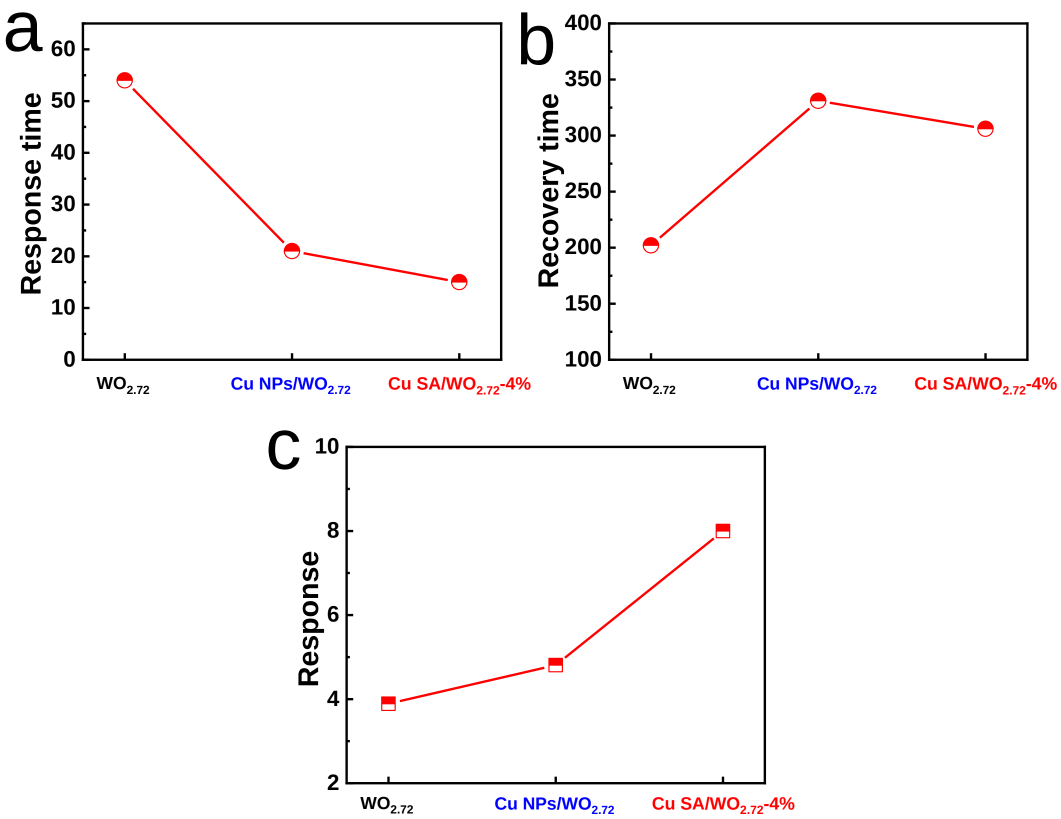


**Figure S22.** (a) Response time and (b) recovery time of pristine $WO_{2.72}$, Cu SA/$WO_{2.72}$-4% and Cu NPs/$WO_{2.72}$ sensors to 2.5 ppm toluene. (c) Response of pristine $WO_{2.72}$, Cu SA/$WO_{2.72}$-4% and Cu NPs/$WO_{2.72}$ sensors to 2.5 ppm toluene.

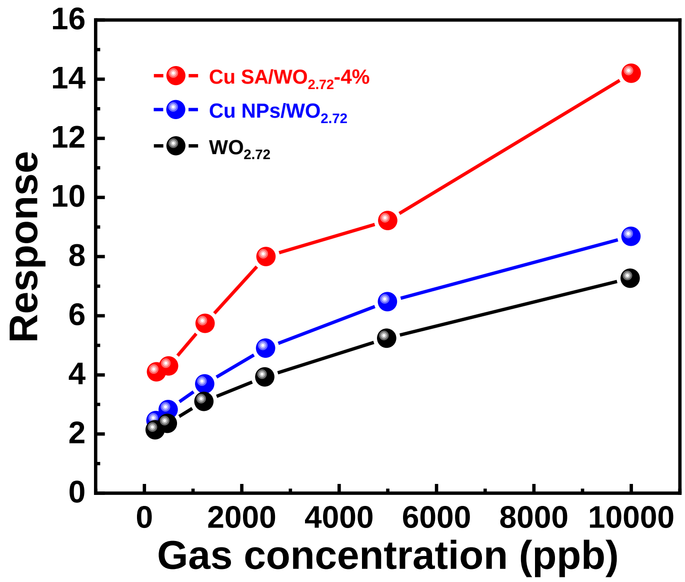


**Figure S23.** The response of pristine $WO_{2.72}$, Cu SA/$WO_{2.72}$-4% and Cu NPs/$WO_{2.72}$ sensors to toluene in different concentrations (250-10000 ppb).

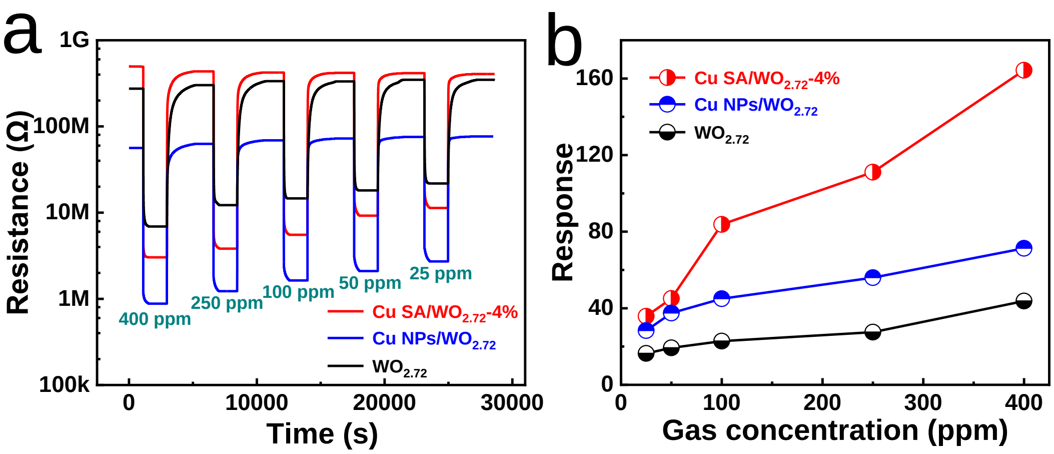


**Figure S24.** (a) Dynamic resistance curves of pristine $WO_{2.72}$, Cu SA/$WO_{2.72}$-4% and Cu NPs/$WO_{2.72}$ sensors toward toluene at different concentrations (25-400 ppm). (b) The relative response versus the toluene concentration illustration of the pristine $WO_{2.72}$, Cu SA/$WO_{2.72}$-4% and Cu NPs/$WO_{2.72}$ sensors.

The response values of pristine $WO_{2.72}$, Cu SA/$WO_{2.72}$-4% and Cu NPs/$WO_{2.72}$ sensors increase with increasing toluene concentration from 25 ppm to 400 ppm. As expected, the Cu SA/$WO_{2.72}$-4% sensor shows relatively high response towards pristine $WO_{2.72}$ and Cu NPs/$WO_{2.72}$.

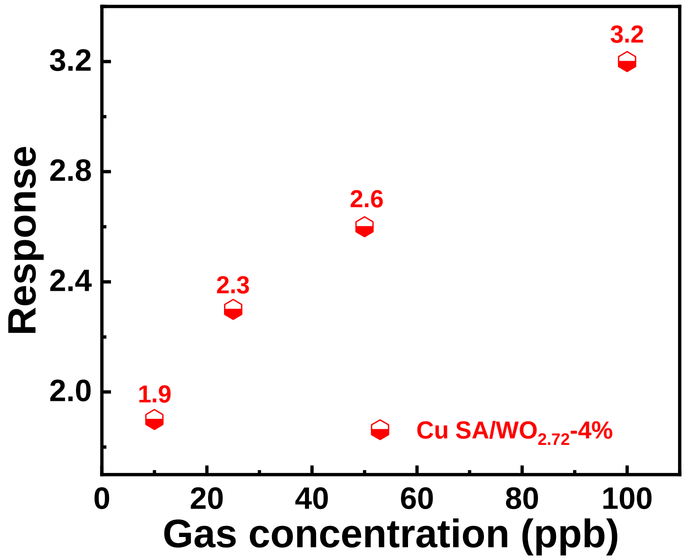


**Figure S25.** Response of Cu SA/$WO_{2.72}$-4% sensor to lower concentration toluene (10, 25, 50 and 100 ppb).

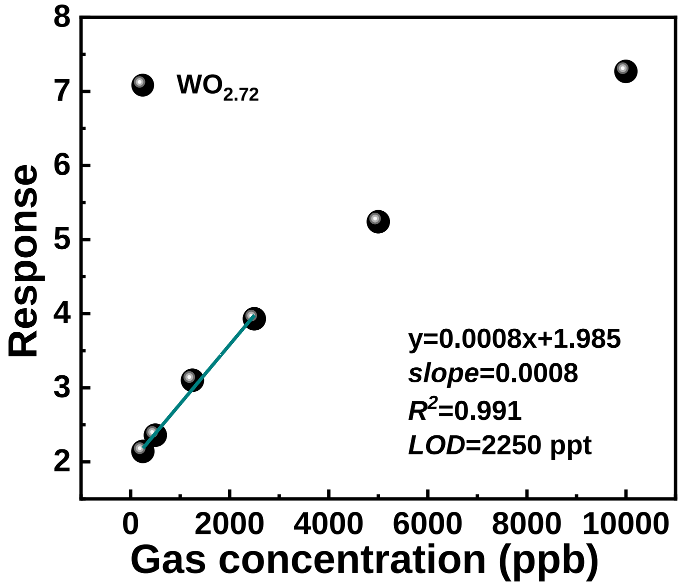


**Figure S26.** Calculation of the limit of detection (LOD). The linear fitting of pristine $WO_{2.72}$ sensor response with toluene concentrations in the linear region, the slope is 0.0008 $ppb^{-1}$.

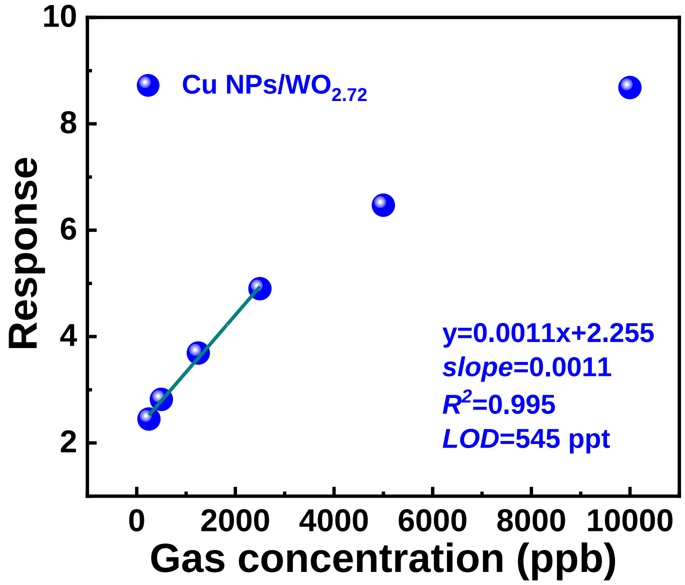


**Figure S27.** Calculation of the limit of detection (LOD). The linear fitting of Cu NPs/$WO_{2.72}$ sensor response with toluene concentrations in the linear region, the slope is 0.0011 $ppb^{-1}$.

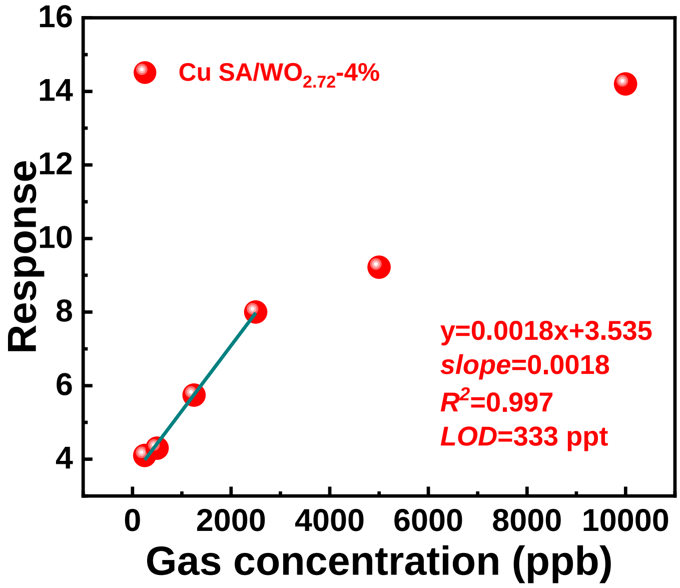


**Figure S28.** Calculation of the limit of detection (LOD). The linear fitting of Cu SA/$WO_{2.72}$-4% sensor response with toluene concentrations in the linear region, the slope is 0.0018 $ppb^{-1}$.

According to the International Union of Pure and Applied Chemistry (IUPAC), the theoretical limit of detection (LOD) of the sensor is defined as: [23,24]

$$LOD(ppb) = 3\frac{RMS_{noise}}{Slope}$$

where $RMS_{noise}$ is the noise of the sensor in the baseline phase, Slope is the slope of the linear region of the sensor response-gas concentration function curve.

We replot 500 data points at the baseline before the toluene exposure and calculate the $RMS_{noise}$ of pristine $WO_{2.72}$, Cu NPs/$WO_{2.72}$ and Cu SA/$WO_{2.72}$-4% sensors to be $6 \times 10^{-4}$, $2 \times 10^{-4}$ and $2 \times 10^{-4}$, respectively. According to LOD calculation equation, the LODs are 2250, 545 and 333 ppt for the pristine $WO_{2.72}$, Cu NPs/$WO_{2.72}$ and Cu SA/$WO_{2.72}$-4% sensors, respectively.

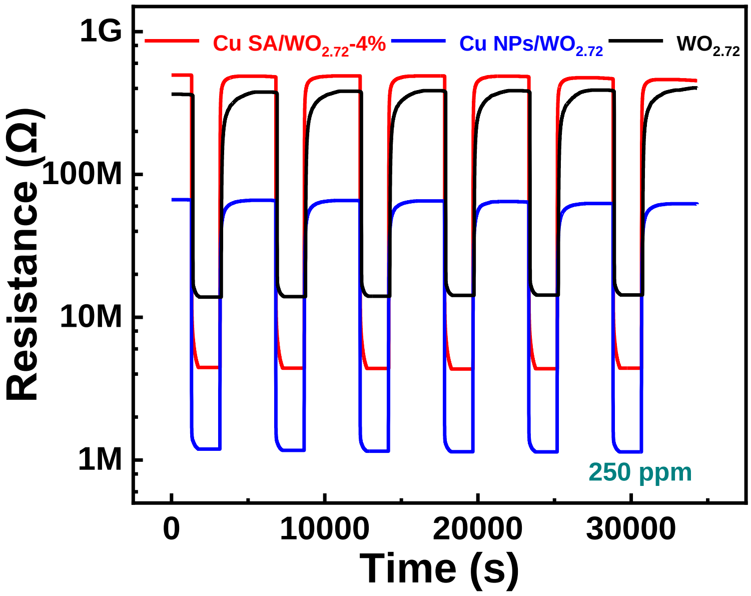


**Figure S29.** Repeatability tests of pristine $WO_{2.72}$, Cu SA/$WO_{2.72}$-4% and Cu NPs/$WO_{2.72}$ sensors to 250 ppm toluene.

The standard deviation formula is as follows: [25,26]

$$\sigma = \sqrt{\frac{\sum_{i=1}^{n}(R_i - \mu)^2}{n}}$$

where $\sigma$ is the standard deviation of sensor response values, $\mu$ is the average value of sensor response values, $R_i$ is the sensor response values, and $n$ is the number of repeated test cycles ($n = 5$).

According to the standard deviation formula, it can be concluded that the standard deviation of response values based on pristine $WO_{2.72}$, Cu NPs/$WO_{2.72}$ and Cu SA/$WO_{2.72}$-4% sensors are 0.04, 0.03 and 0.01, respectively.

The attenuation ratio (α) of the response signal is defined as: [27,28]

$$\alpha = \frac{R_{initial} - R_{final}}{R_{initial}}$$

where $R_{initial}$ and $R_{final}$ are the initial and final response values of the sensor, respectively. According to above-mentioned calculation equation, the attenuation ratios (α) are 15.2%, 9.6% and 4.8% for pristine $WO_{2.72}$, Cu NPs/$WO_{2.72}$ and Cu SA/$WO_{2.72}$-4% sensors, respectively.

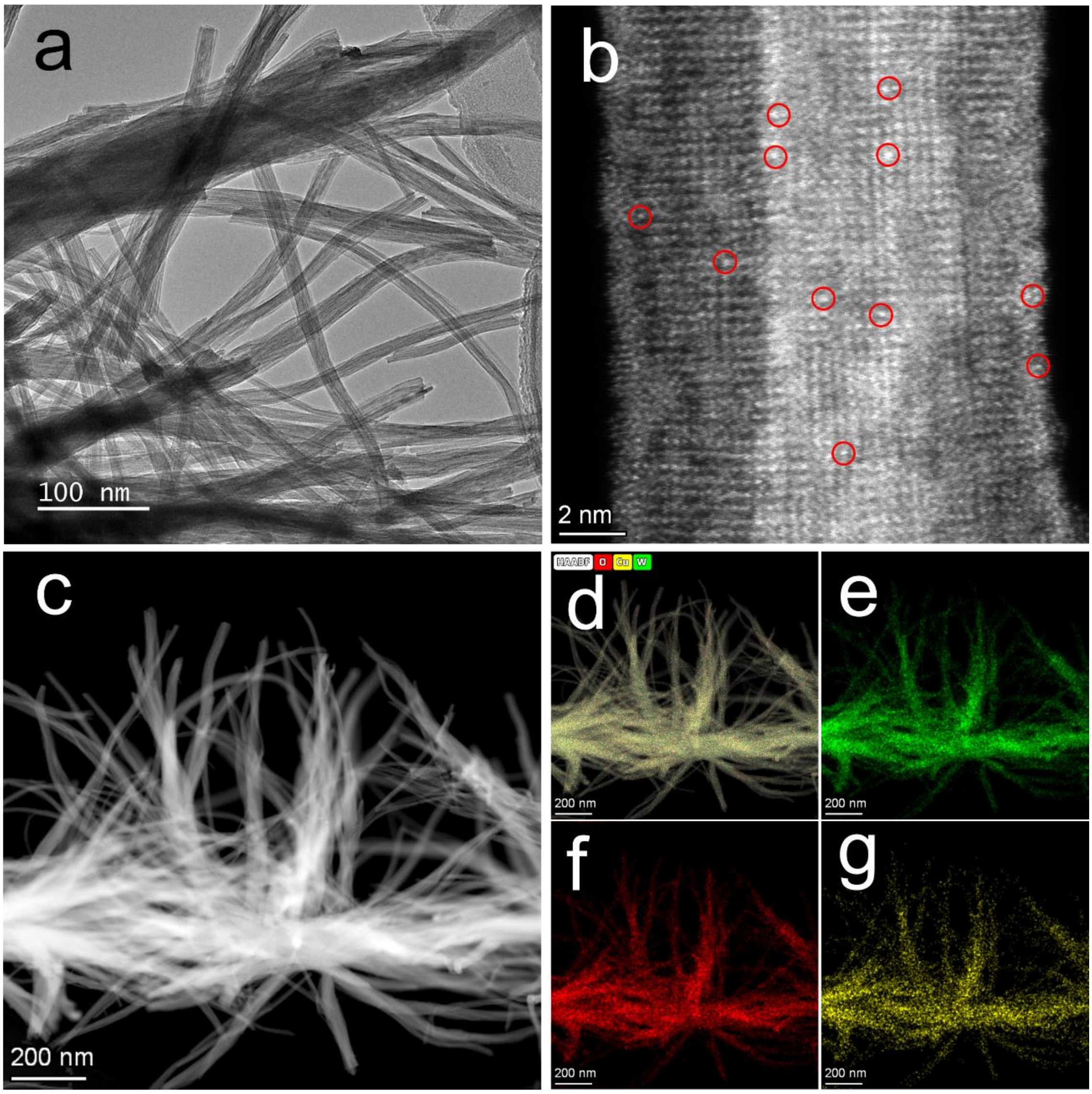


**Figure S30.** Characterization of sensitive material after the continuous gas sensing test to 2.5 ppm toluene gas for thirty days based on the Cu SA/$WO_{2.72}$-4% sensor. (a) TEM image, (b) AC HAADF-STEM image, (c) AC HAADF-STEM image and (d-g) corresponding EDX elemental mapping for elements W, Cu and O.

The morphology of sensitive material keeps the same after the continuous gas sensing test to 2.5 ppm toluene gas for thirty days based on the Cu SA/$WO_{2.72}$-4% sensor, exhibiting bundle-like nanowires, demonstrating the outstanding stability of Cu SA/$WO_{2.72}$-4% sample. It can be distinctly observed that Cu atoms are still in isolated status. No agglomeration of Cu species happens during the toluene gas sensing test. Meanwhile, the W, Cu and O elements are still uniformly dispersed. Corresponding characterizations verify that no morphology change occurs and Cu species remain in monodispersion on it with negligible loss. From this data, it is evident

that the Cu SA/$WO_{2.72}$-4% sample has sufficient potential to be developed as a reliable toluene gas sensor owing to its excellent stability.

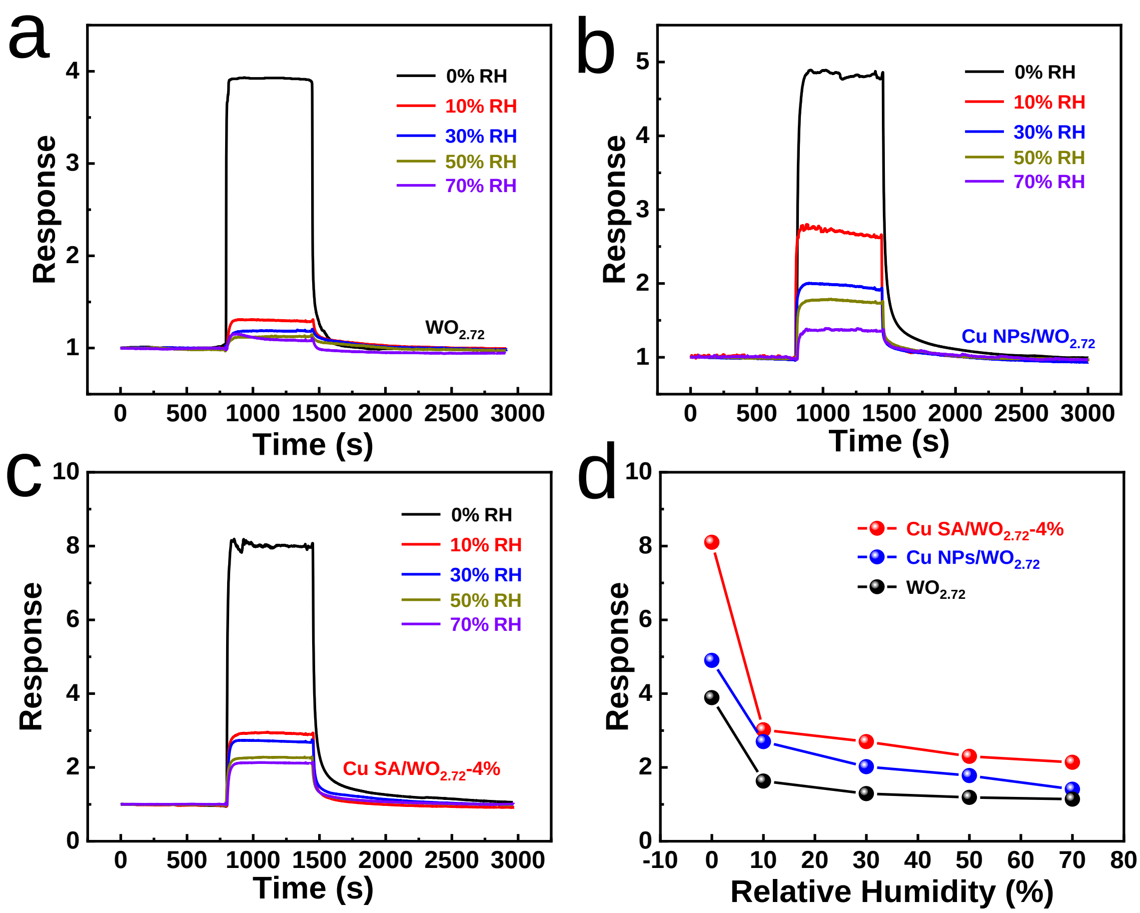


**Figure S31.** Real-time sensing curves of (a) pristine $WO_{2.72}$, (b) Cu NPs/$WO_{2.72}$, (c) Cu SA/$WO_{2.72}$-4% sensors to 2.5 ppm toluene at different relative humidity (RH), and (d) the relationship between response and RH.

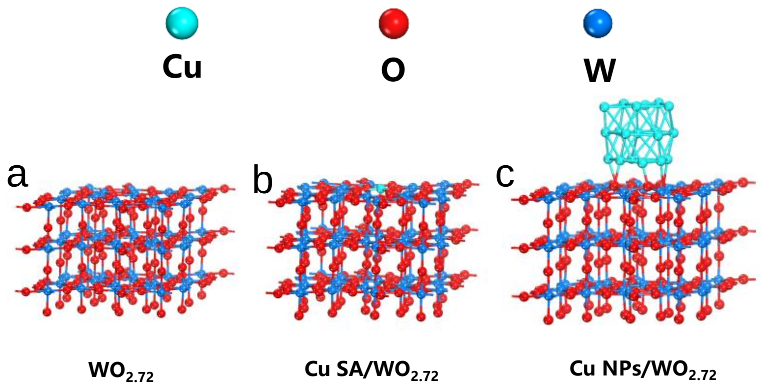


**Figure S32.** The crystal structure models are established for (a) pristine $WO_{2.72}$, (b) Cu SA/$WO_{2.72}$ and (c) Cu NPs/$WO_{2.72}$.

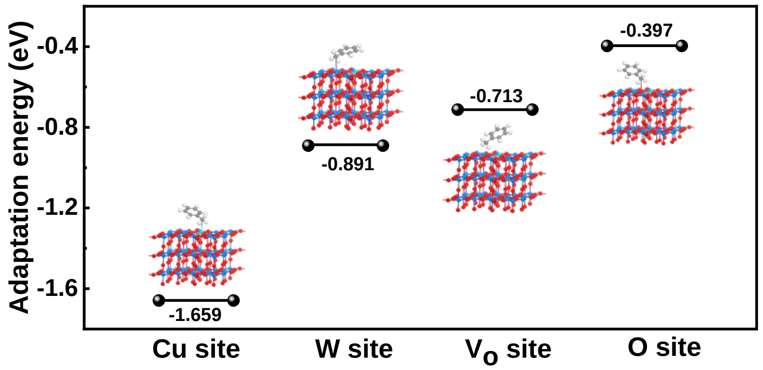


**Figure S33.** The adsorption energy ($E_{ads}$) of Cu site, W site, oxygen vacancy ($V_o$) site and O site toward toluene gas molecule on Cu SA/$WO_{2.72}$ crystal structure.

Based on experimental results and previous studies, the optimum crystal structure model for Cu SA/$WO_{2.72}$ is established, with the $WO_{2.72}$ supported Cu NPs and pristine $WO_{2.72}$ as a control. To acquire the optimum active site of Cu SA/$WO_{2.72}$ crystal structure for toluene molecule, the adsorption energies ($E_{ads}$) of toluene molecule at Cu site, W site, O site and oxygen vacancy ($V_o$) site are evaluated. The Cu site has the lowest adsorption energy with a value of -1.659 eV, compared to -0.891, -0.397 and -0.713 eV of the W site, O site and oxygen vacancy ($V_o$) site, respectively. Consequently, the toluene molecules are preferentially absorbed on Cu site, indicating that the Cu atom is the best active site for toluene oxidation reaction. Next, for Cu SA/$WO_{2.72}$, other calculations are performed based on the Cu site.

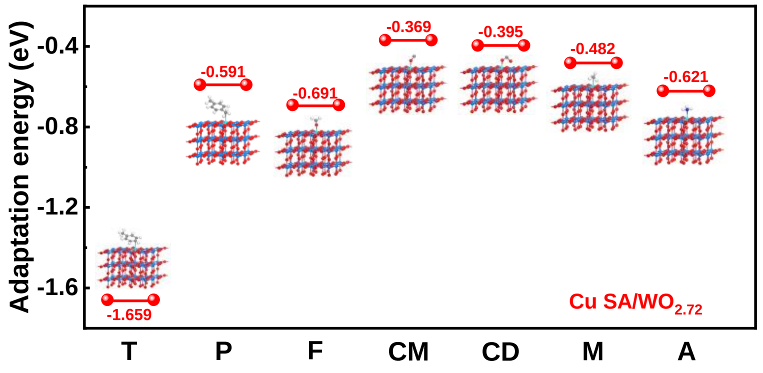


**Figure S34.** The adsorption energies ($E_{ads}$) of Cu SA/$WO_{2.72}$ crystal structure toward different gas molecules (T is toluene, P is paraxylene, F is formaldehyde, M is methane, CM is carbon monoxide, CD is carbon dioxide and A is ammonia).

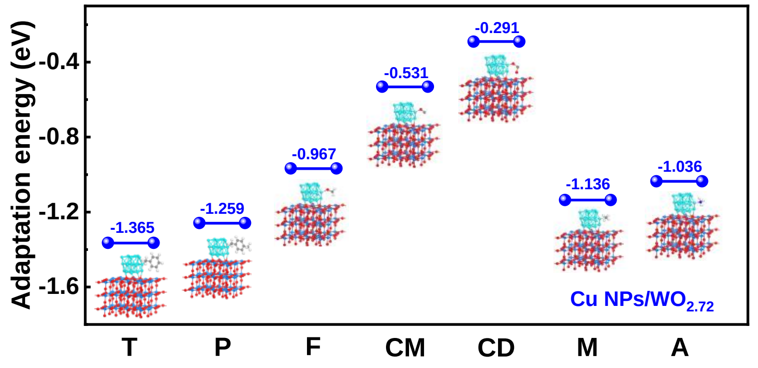


**Figure S35.** The adsorption energies ($E_{ads}$) of Cu NPs/$WO_{2.72}$ crystal structure toward different gas molecules (T is toluene, P is paraxylene, F is formaldehyde, M is methane, CM is carbon monoxide, CD is carbon dioxide and A is ammonia).

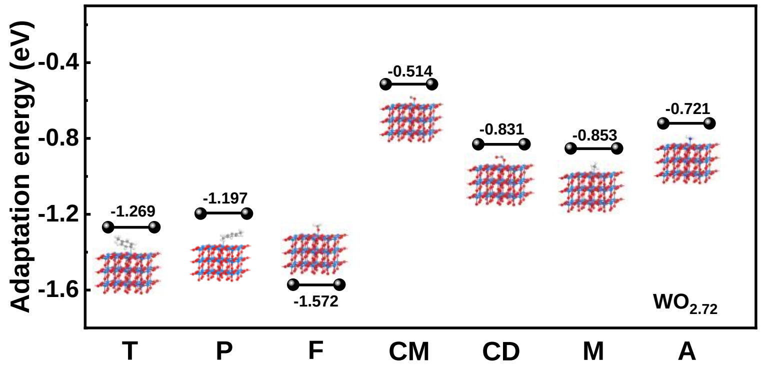


**Figure S36.** The adsorption energies ($E_{ads}$) of pristine $WO_{2.72}$ crystal structure toward different gas molecules (T is toluene, P is paraxylene, F is formaldehyde, M is methane, CM is carbon monoxide, CD is carbon dioxide and A is ammonia).

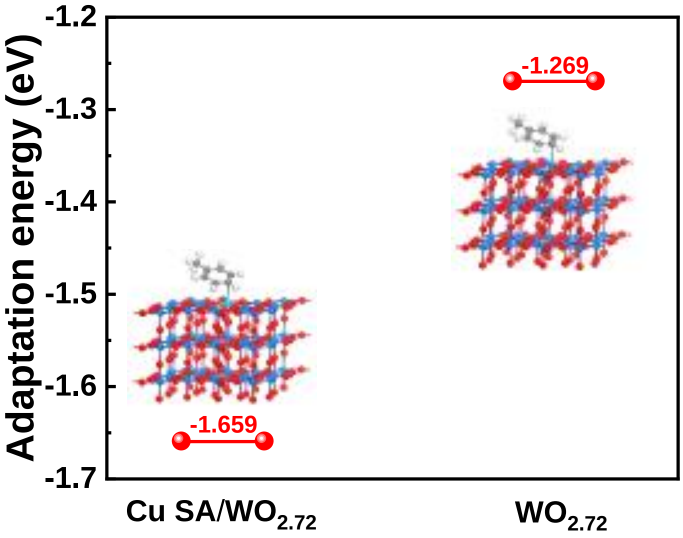


**Figure S37.** The adsorption energies ($E_{ads}$) of toluene on the pristine $WO_{2.72}$ and Cu SA/$WO_{2.72}$.

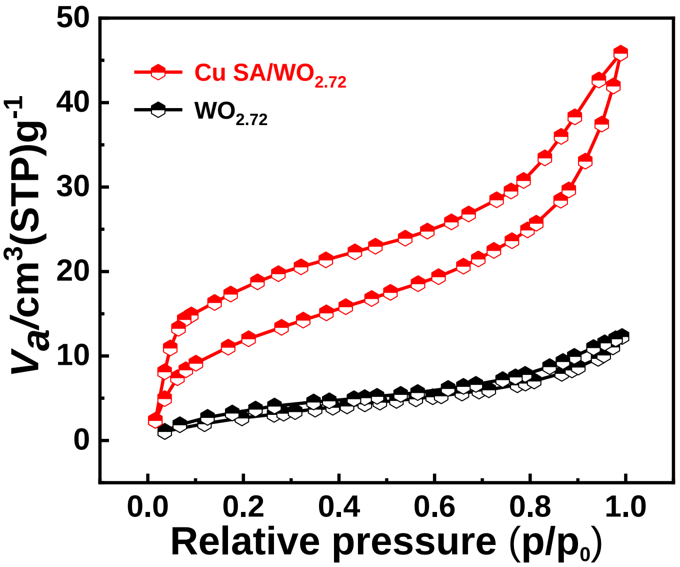


**Figure S38.** Comparative analysis of toluene adsorption-desorption isotherms to pristine $WO_{2.72}$ and Cu SA/$WO_{2.72}$.

The adsorption energy ($E_{ads}$) of -1.659 eV for toluene on Cu SA/$WO_{2.72}$ is lower than that of pristine $WO_{2.72}$ (-1.269 eV), which exhibits the interaction between toluene and $WO_{2.72}$ could be improved by Cu SAs. As illustrated in Figure S38, the toluene absorption capacity of Cu SA/$WO_{2.72}$ sample increases significantly. These results indicate that more toluene molecules react with reactive oxygen species on the surface of Cu SA/$WO_{2.72}$, leading to enhanced gas sensing performance.

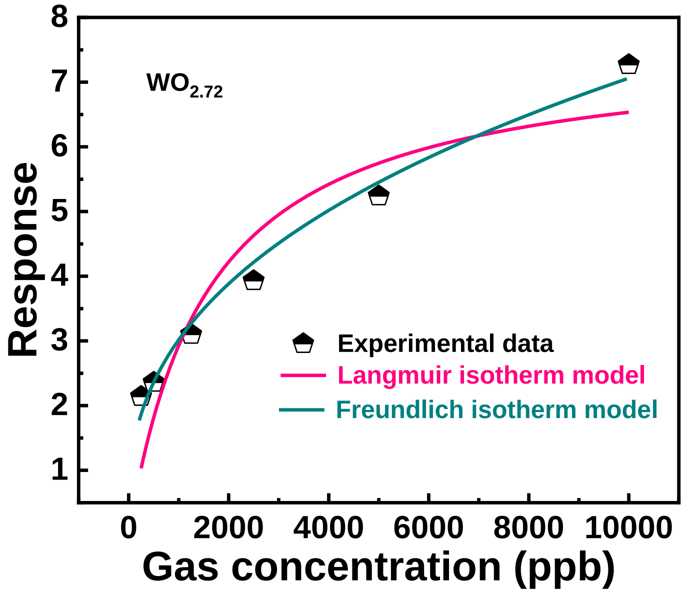


**Figure S39.** Fitting of Langmuir and Freundlich isothermal model to pristine $WO_{2.72}$ sensor response.

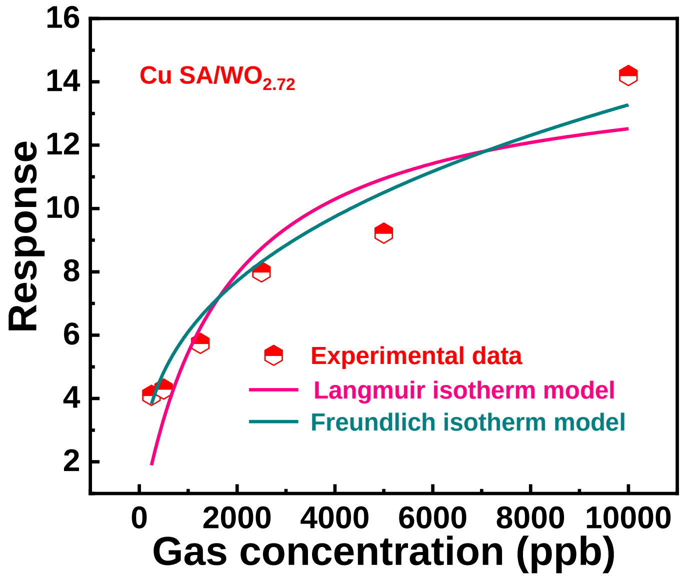


**Figure S40.** Fitting of Langmuir and Freundlich isothermal model to Cu SA/$WO_{2.72}$ sensor response.

The gas sensing process of pristine $WO_{2.72}$ and Cu SA/$WO_{2.72}$ sensors is the adsorption-reaction-desorption behavior of gas molecules on the sensor film. We acquire the correlation coefficient ($R^2$) by fitting the isothermal response models.

Langmuir isothermal model: [29]

$$\mu = \frac{R_i}{R_{max}} = \frac{K_L C}{1 + K_L C}$$

$$\frac{C}{R_i} = \frac{C}{R_{max}} + \frac{1}{K_L R_{max}}$$

where $R_i$ is sensor response to toluene with a certain concentration of $C$ (ppb), $K_L$ and $R_{max}$ are equilibrium constant and maximum theoretical response of the sensor, respectively.

Freundlich isothermal model: [30]

$$R_i = K_F C^{1/n}$$

$$\ln R_i = \ln K_F + \frac{1}{n}\ln C$$

where $K_F$ is equilibrium constant, the larger the $K_F$ value, the better the sensor's performance, $\frac{1}{n}$ is empirical constant associated with sensor response.

For the Freundlich model, the correlation coefficients ($R^2$) of pristine $WO_{2.72}$ and Cu SA/$WO_{2.72}$ sensors are 0.984 and 0.953, respectively, both of which exceed those obtained from the Langmuir model. Thus, the isothermal response models of those sensors belong to the Freundlich model. These results indicate that the inhomogeneity of sensing material surface leads to the different affinity for gas molecules. Thus, a toluene molecule may be adsorbed by different active sites. The equilibrium constant ($K_F$) is calculated based on the Freundlich isothermal model, assuming that the amount of the surface-adsorbed gas is directly proportional to the sensor response. The equilibrium constants ($K_F$) of pristine $WO_{2.72}$ and Cu SA/$WO_{2.72}$ sensors are 0.22 and 0.59, respectively.

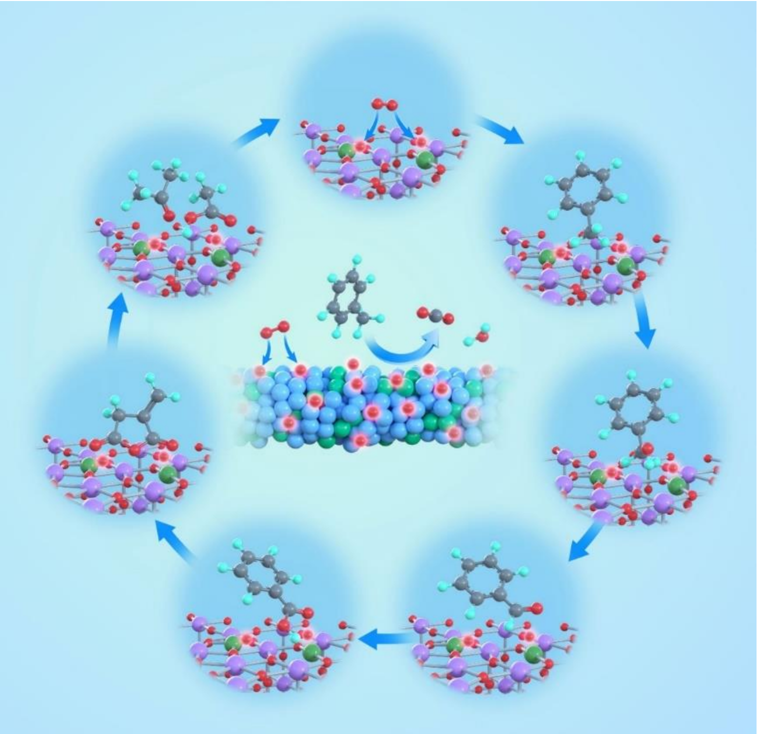

**Figure S41.** Proposed toluene sensing and conversion mechanism on Cu SA/$WO_{2.72}$-based sensor.

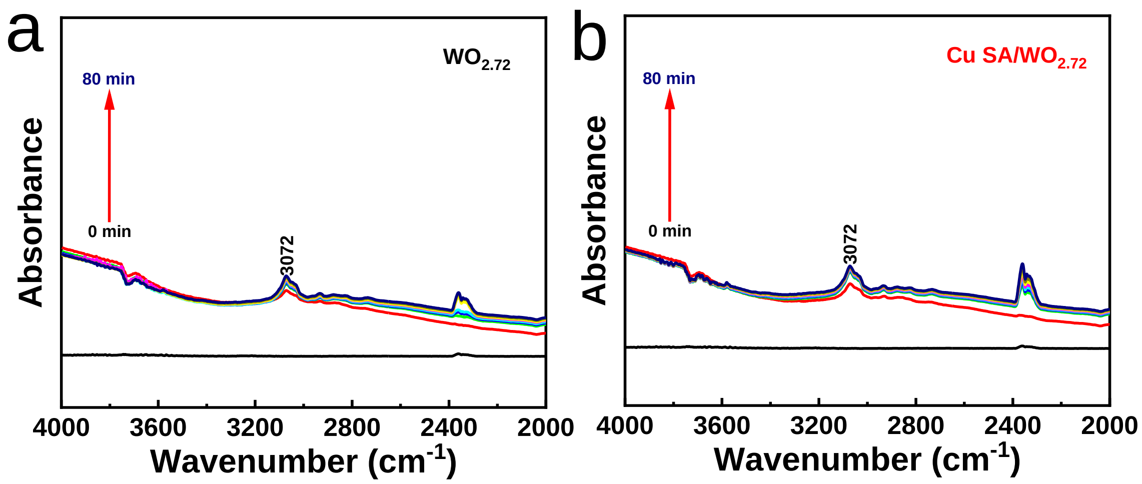


**Figure S42.** In situ DRIFTS spectra (2000-4000 $cm^{-1}$) for the oxidation of toluene over (a) pristine $WO_{2.72}$ and (b) Cu SA/$WO_{2.72}$ samples at different reaction time.

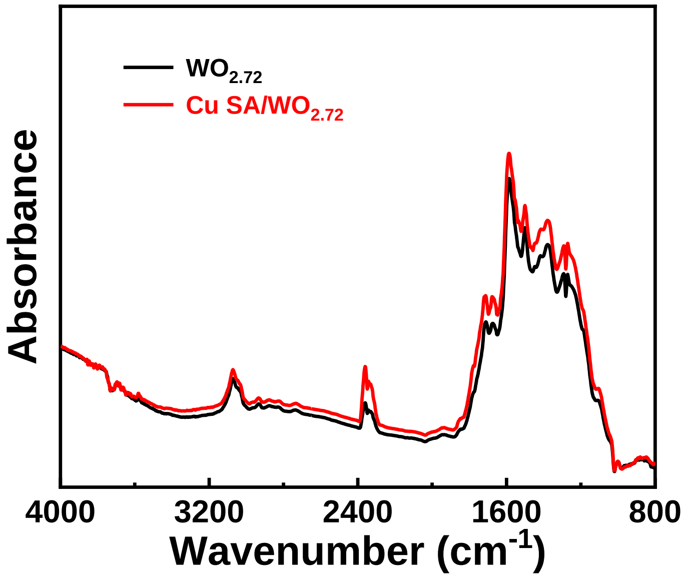


**Figure S43.** In situ DRIFTS spectra for the oxidation of toluene over pristine $WO_{2.72}$ and Cu SA/$WO_{2.72}$ samples at 80 mins.

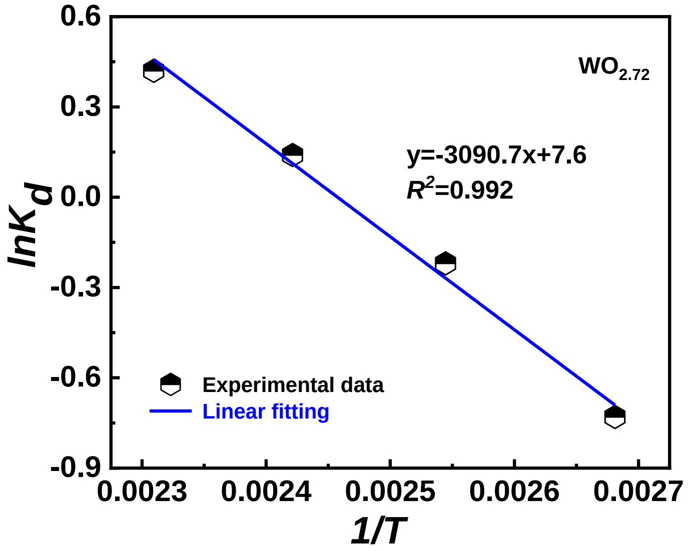


**Figure S44.** Fitting of thermodynamic equation to pristine $WO_{2.72}$ sensor response.

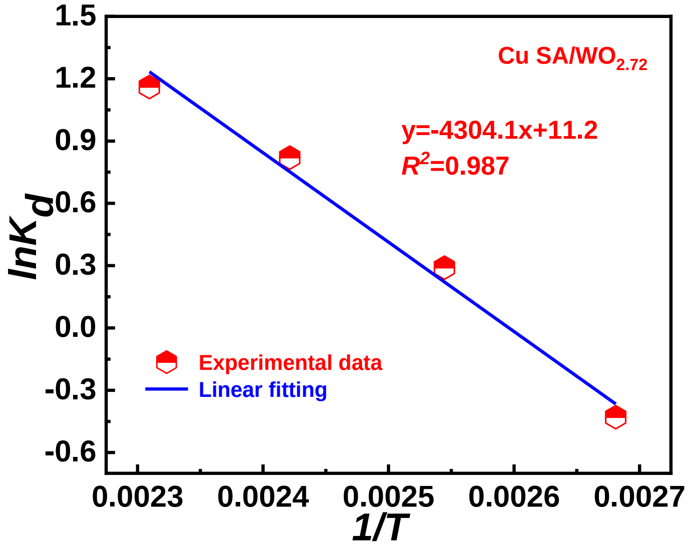


**Figure S45.** Fitting of thermodynamic equation to Cu SA/$WO_{2.72}$ sensor response.

Thermodynamic equation of response: [29,30]

$$K_d = \frac{R_i}{C}$$

$$\mathrm{In}K_d = \frac{\Delta S^0}{R_\alpha} - \frac{\Delta H^0}{R_\alpha T}$$

The Gibbs free energy ($\Delta G^0$) of the sensor is calculated as follows:

$$\Delta G^0 = \Delta H^0 - T\Delta S^0$$

Where $R_i$ is sensor response, $C$ is the gas concentration ($C$ = 2.5 ppm), $\Delta H^0$ is enthalpy change, $\Delta S^0$ is entropy change, $\Delta G^0$ is gibbs free energy change, $T$(K) is kelvin temperature, and $R_\alpha$ is the Avogadro constant, 8.314 J $mol^{-1}$ $K^{-1}$.

The amount of the surface-adsorbed gas is directly proportional to the sensor response. Here, the sensor response values are fitted by thermodynamic equation. The values of $\Delta H^0$ and $\Delta S^0$ are calculated from the slope and intercept, respectively. As shown in Table S8, $\Delta H^0$>0 and $\Delta G^0$<0 (433 K) indicate that the gas sensing process is endothermic and spontaneous.

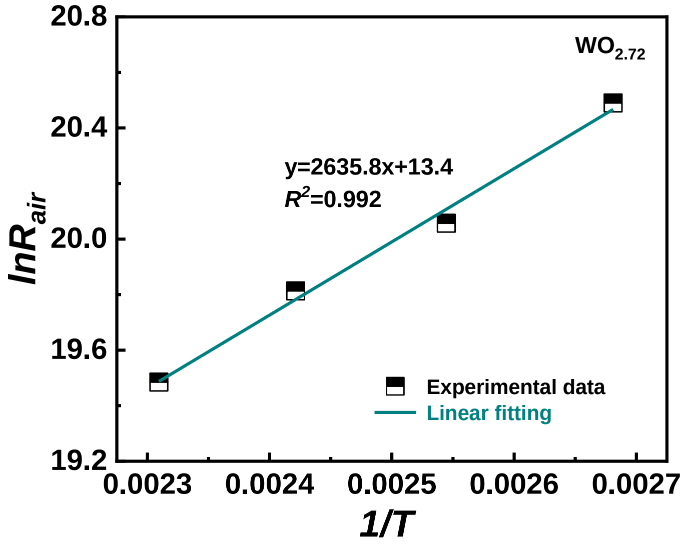


**Figure S46.** Arrhenius plot of log resistance versus reciprocal temperature for pristine $WO_{2.72}$ sensor.

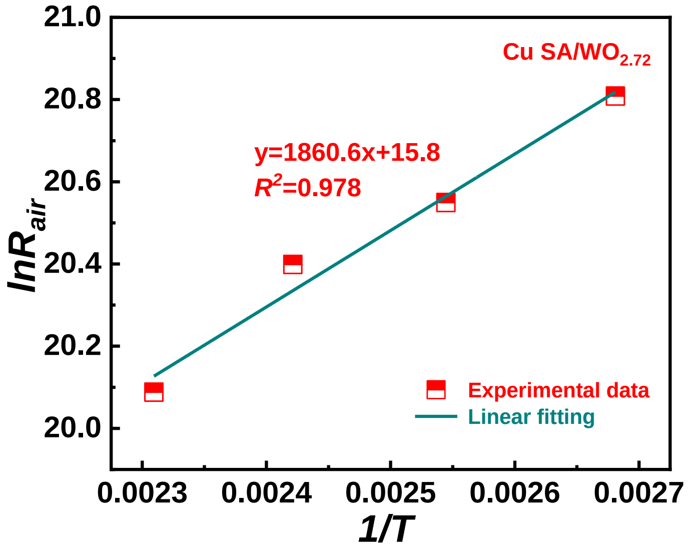


**Figure S47.** Arrhenius plot of log resistance versus reciprocal temperature for Cu SA/$WO_{2.72}$ sensor.

As we all know, the number of carriers in semiconductor increases exponentially with temperature. The higher the temperature, the higher the number of carriers, the higher the conductivity. In addition, the basic characteristics of semiconductor gas sensor resistance are temperature-dependent, which is closely related to the conduction mechanism of semiconductor materials. Thus, the sensor resistance decreases exponentially with temperature, satisfying the following the Arrhenius relation: [31,32]

$$R_{air} = R_0 \exp \frac{E_A}{K_B T}$$

$$\ln R_{air} = \frac{E_A}{K_B T} + C_0$$

where $K_B$ is the Boltzmann constant, T is absolute temperature in degrees Kelvin, $E_A$ is charge transport activation energy, $R_{air}$ is resistance value of sensor and $C_0$ is a constant related to the $R_0$.

The charge transport activation energies ($E_A$) for pristine $WO_{2.72}$ and Cu SA/$WO_{2.72}$ sensors are estimated from the slop of the Arrhenius plots acquired from their conductivity versus temperature characteristics. The $E_A$ values for the pristine $WO_{2.72}$ and Cu SA/$WO_{2.72}$ sensors in the temperature range of 100-160°C are 0.23 eV and 0.16 eV, respectively.

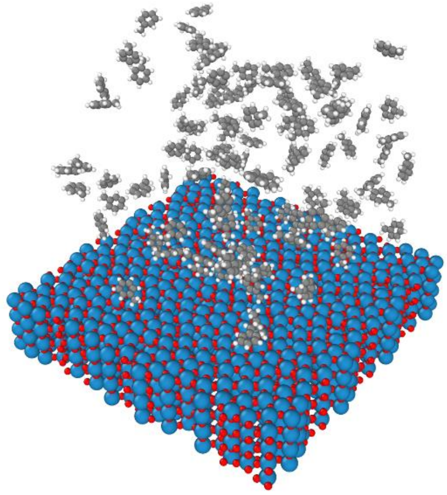

**Figure S48.** MD simulation snapshot for the dynamic process of toluene diffusion on pristine $WO_{2.72}$ sample.

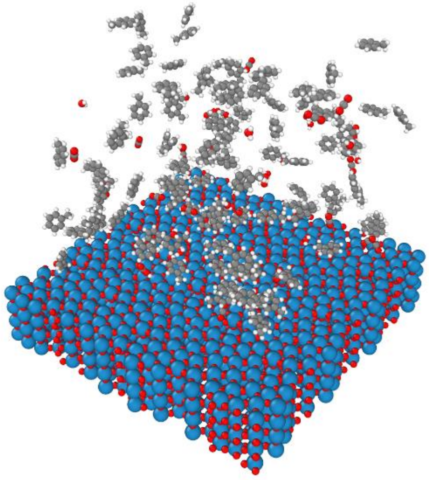

**Figure S49.** MD simulation snapshot for the dynamic process of carbon dioxide and water vapor diffusion on pristine $WO_{2.72}$ sample.

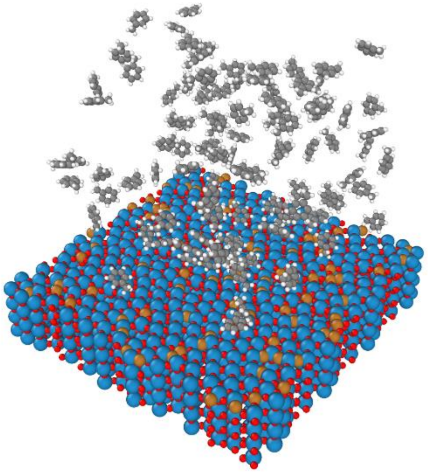

**Figure S50.** MD simulation snapshot for the dynamic process of toluene diffusion on Cu SA/$WO_{2.72}$ sample.

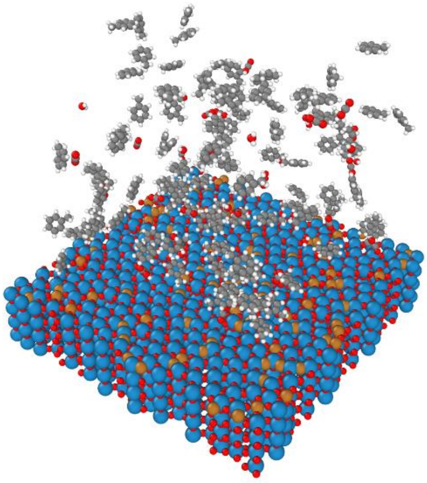

**Figure S51.** MD simulation snapshot for the dynamic process of carbon dioxide and water vapor diffusion on Cu SA/$WO_{2.72}$ sample.

**Table S1.** Textual properties of pristine $WO_{2.72}$, Cu SA/$WO_{2.72}$-4%, Cu SA/$WO_{2.72}$-6% and Cu SA/$WO_{2.72}$-8% samples.

| Sample | BET Surface Area ($m^2 \cdot g^{-1}$) | Average pore size (nm) | Micropore Area ($m^2 \cdot g^{-1}$) |
| --- | --- | --- | --- |
| Pristine $WO_{2.72}$ | 78.57 | 6.93 | 36.35 |
| Cu SA/$WO_{2.72}$-4% | 98.72 | 6.21 | 29.90 |
| Cu SA/$WO_{2.72}$-6% | 89.48 | 5.66 | 41.45 |
| Cu SA/$WO_{2.72}$-8% | 85.00 | 8.06 | 23.43 |

**Table S2.** EXAFS fitting parameters at the Cu K-edge for Cu SA/$WO_{2.72}$-4% ($S_0^2$=0.91).

| Sample | Shell | CN[a] | R(Å)[b] | $\sigma^2$ ($Å^2 \cdot 10^{-3}$)[c] | $\Delta E_0$ (eV)[d] | R factor (%) |
|---|---|---|---|---|---|---|
| Cu K-edge | | | | | | |
| Cu foil | Cu-Cu | 12* | 2.54±0.004 | 0.0087 | 3.76±0.61 | 0.4 |
| Cu SA/$WO_{2.72}$-4% | Cu-O | 3.6±0.3 | 1.96±0.020 | 0.0033 | 4.53±1.77 | 1.3 |

[a]CN is the coordination number; [b]R is the distance between absorber and backscatter atoms; [c]$\sigma^2$ is the debye-Waller factor to account for both thermal and structural disorders; [d]$\Delta E_0$ is the inner potential correction; R is the factor indicates the goodness of the fit; $S_0^2$ was fixed to 0.91 according to the experimental EXAFS fit of Cu foil by fixing CN as the known crystallographic value. Fitting range: $3.0 \leq k(/Å) \leq 10$ and $1.5 \leq R(Å) \leq 3.2$ (Cu foil); $3.0 \leq k(/Å) \leq 11.0$ and $1.0 \leq R(Å) \leq \sim 2.6$ (Cu SA/$WO_{2.72}$-4%). A reasonable range of EXAFS fitting parameters: $0.700 < S_0^2 < 1.000$; CN > 0; $\sigma^2 > 0$ $Å^2$; $\Delta E_0 < 10$ eV; R factor < 0.02.

**Table S3.** The peak area ratio of $O_{latt}$, $O_{sur}$ and $O_{ads}$ for pristine $WO_{2.72}$, Cu SA/$WO_{2.72}$-4%, Cu SA/$WO_{2.72}$-6% and Cu SA/$WO_{2.72}$-8% samples.

| Sample | $O_{latt}$ | $O_{sur}$ | $O_{ads}$ |
|---|---|---|---|
| Pristine $WO_{2.72}$ | 71.9% | 20.4% | 7.7% |
| Cu SA/$WO_{2.72}$-4% | 65.3% | 28.4% | 6.3% |
| Cu SA/$WO_{2.72}$-6% | 66.2% | 27.1% | 6.7% |
| Cu SA/$WO_{2.72}$-8% | 66.4% | 26.2% | 7.4% |

**Table S4.** The peak area ratio of $W^{5+}$ and $W^{6+}$ for pristine $WO_{2.72}$, Cu SA/$WO_{2.72}$-4%, Cu SA/$WO_{2.72}$-6% and Cu SA/$WO_{2.72}$-8% samples.

| Sample | $W^{5+}$ peak | $W^{6+}$ peak |
|---|---|---|
| Pristine $WO_{2.72}$ | 12.0% | 88.0% |
| Cu SA/$WO_{2.72}$-4% | 20.0% | 80.0% |
| Cu SA/$WO_{2.72}$-6% | 14.1% | 85.9% |
| Cu SA/$WO_{2.72}$-8% | 12.7% | 87.3% |

**Table S5.** Properties comparison of toluene sensors.

| Materials | Tem. | Con. | Ra/Rg | Actual detection concentration | Refs. |
|---|---|---|---|---|---|
| Au-$MoO_3$ | 250°C | 5 ppm | 2.5 | 0.1 ppm | [33] |
| C-$WO_3$ | 320°C | 100 ppb | 1.8 | 0.1 ppm | [34] |
| Mn-$Co_3O_4$ | 280°C | 5 ppm | ~6.5 | 5 ppm | [35] |
| ZnO-$CeO_2$ | 200°C | 10 ppm | ~8.8 | 10 ppb | [36] |
| $Co_3O_4$-DNWs | 210°C | 100 ppm | 11.76 | 50 ppm | [37] |
| CuO-$Ti_3C_2T_x$ | 250°C | 50 ppm | 11.4 | 10 ppm | [38] |
| $NiGa_2O_4$-NiO | 230°C | 5 ppm | ~4.0 | 0.5 ppm | [39] |
| MWCNTs | 150°C | 5 ppm | 5.06 | 1 ppm | [40] |
| $Zn_2SnO_4$ | 280°C | 20 ppm | 6.1 | 5 ppm | [41] |
| $ZnFe_2O_4$ | 250°C | 0.2 ppm | 1.34 | 0.2 ppm | [42] |
| $NiFe_2O_4$ | 260°C | 100 ppm | 6.41 | 1 ppm | [43] |
| $NiFe_2O_4$ | 200°C | 5 ppm | ~1.9 | 1 ppm | [44] |
| $Co_3O_4$ | 180°C | 200 ppm | 8.5 | 5 ppm | [45] |
| NiO | 210°C | 200 ppm | 1.6 | 100 ppm | [46] |
| $Ni_{0.33}Co_{0.67}Fe_2O_4$ | 300°C | 10 ppm | 5.37 | 10 ppm | [47] |
| **Cu SA/$WO_{2.72}$** | **160°C** | **10 ppb** | **1.9** | **10 ppb** | **This work** |
| **Cu SA/$WO_{2.72}$** | **160°C** | **2.5 ppm** | **8.1** | **10 ppb** | **This work** |

The Temperature is denoted by Tem.; The Concentration is denoted by Con.; The References is denoted by Refs..

**Table S6.** Infrared vibration information of toluene related to intermediate species for the oxidation over pristine $WO_{2.72}$ and Cu SA/$WO_{2.72}$ samples. [48-50]

| Wavenumber ($cm^{-1}$) | Vibration mode |
|---|---|
| 1035, 1108, 1184 | The C-O stretching vibration of benzyl alcohol |
| 1587, 1672 | The C=O vibration peak subordinate to benzaldehyde |
| 1498 | The anti-symmetric vibration of the COO-group of the benzoate |
| 1294, 1268, 1375, 1712 | The vibrational peak of maleic anhydride |
| 1672 | the C=O stretching vibration |
| 1419 | the O-H bending of the carboxyl group |
| 1448 | the characteristic stretching vibration of the benzene skeleton |
| 3072 | the unsaturated =C-H bond stretching vibration. |

**Table S7.** Langmuir and Freundlich response model parameters of sensor.

| Response isothermal model | Langmuir mode | | | Freundlich mode | | |
|---|---|---|---|---|---|---|
| | $R^2$ | $R_{max}$ | $K_L$ | $R^2$ | $\frac{1}{n}$ | $K_F$ |
| Pristine $WO_{2.72}$ | 0.850 | 7.57 | $6.29\times10^{-4}$ | 0.984 | 0.38 | 0.22 |
| Cu SA/$WO_{2.72}$ | 0.830 | 14.62 | $5.94\times10^{-4}$ | 0.953 | 0.34 | 0.59 |

**Table S8.** Thermodynamic parameters of sensor.

| Sensor | ΔH° (KJ·mol$^{-1}$) | ΔS° [J·(K·mol)$^{-1}$] | ΔG° (J·mol$^{-1}$) | | | |
|---|---|---|---|---|---|---|
| | | | 373K | 393K | 413K | 433K |
| Pristine $WO_{2.72}$ | 25.7 | 63.2 | 2126.4 | 862.4 | -401.6 | -1665.6 |
| Cu SA/$WO_{2.72}$ | 35.8 | 93.1 | 1073.7 | -788.3 | -2650.3 | -4512.3 |

**References**

[1] G. Kresse, J. Furthmüller, *Comput. Mater. Sci.* **1996**, 6, 15-50.

[2] G. Kresse, J. Furthmüller, *Phys. Rev. B* **1996**, 54, 11169-11186.

[3] J. P. Perdew, K. Burke, M. Ernzerhof, *Phys. Rev. Lett.* **1996**, 77, 3865-3868.

[4] G. Kresse, D. Joubert, *Phys. Rev. B* **1999**, 59, 1758-1775.

[5] P. E. Blöchl, *Phys. Rev. B* **1994**, 50, 17953-17979.

[6] W. G. Hoover, *Phys. Rev. A: At. Mol. Opt. Phys.* **1985**, 31, 1695-1697.

[7] W. L. Jorgensen, J. Chandrasekhar, J. D. Madura, R.W. Impey, M. L. Klein, *J. Chem. Phys.* **1983**, 79, 926-935.

[8] E. K. Watkins, W. L. Jorgensen, *J. Phys. Chem. A* **2001**, 105, 4118-4125.

[9] S. Plimpton, *J. Comput. Phys.* **1995**, 117, 1-19.

[10] W. W. Sun, Y. J. Li, Y. M. Liu, Q. P. Guo, S. Q. Luo, J. G. Yang, C. M. Zheng, K. Xie, *J. Mater. Chem. A* **2018**, 6, 14155-14161.

[11] S. Cong, Y. Y. Yuan, Z. G. Chen, J. Y. Hou, M. Yang, Y. L. Su, Y. Y. Zhang, L. Li, Q. W. Li, F. X. Geng, Z. G. Zhao, *Nat. Commun.* **2015**, 6, 7800.

[12] W. F. Xiong, D. H. Si, J. D. Yi, Y. B. Huang, H. F. Li, R. Cao, *Appl. Catal. B-Environ.* **2022**, 314, 121498.

[13] W. S. Zhang, Y. Fan, T. W. Yuan, B. Lu, Y. M. Liu, Z. X. Li, G. J. Li, Z. X. Cheng, J. Q. Xu, ACS *Appl. Mater. Interfaces* **2020**, 12, 3755-3763.

[14] S. Z. Wang, Y. Wang, Y. C. Song, X. H. Jia, J. Yang, Y. Li, J. X. Liao, H. J. Song, *Energy Storage Mater.* **2021**, 43, 422-429.

[15] K. K. Patra, Z. Liu, H. Lee, S. Hong, H. Song, H. G. Abbas, Y. Kwon, S. Ringe, J. Oh, *ACS Catal.* **2022**, 12, 10973-10983.

[16] M. Y. Sun, Y. F. Zhang, W. Liu, X. P. Zhao, H. Luo, G. Miao, Z. Q. Wang, S. G. Li, L. Z. Kong, *Green Chem.* **2022**, 24, 9489-9495.

[17] H. B. Yin, Z. Chen, Y. Peng, S. C. Xiong, Y. D. Li, H. Yamashita, J. H. Li, *Angew. Chem. Int. Ed.* **2022**, 61, 202114242.

[18] X. F. Hu, X. Li, H. M. Yang, C. J. Xu, W. Q. Xiong, X. Guo, C. S. Xie, D. W. Zeng, *ACS Sens.* **2022**, 7, 1894-1902.

[19] C. J. Dong, R. N. Tian, Y. L. Zhang, K. L. Liu, G. Chen, H. T. Guan, Z. Y. Yin, *Chem. Eng. J.* **2022**, 442, 136094.

[20] G. Xi, J. Ye, Q. Ma, N. Su, H. Bai, C. Wang, *J. Am. Chem. Soc.* **2012**, 134, 6508-6511.

[21] X. Y. Liu, P. F. Wang, Y. Li, S. H. Zhan, *Chem. Eng. J.* **2022**, 431, 134134.

[22] H. Song, Y. G. Li, Z. R. Lou, M. Xiao, L. Hu, Z. Z. Ye, L. P. Zhu, *Appl. Catal. B-Environ.* **2015**, 166-167, 112-120.

[23] V. Dua, S. P. Surwade, S. Ammu, S. R. Agnihotra, S. Jain, K. E. Roberts, S. Park, R. S. Ruoff, S. K. Manohar, *Angew. Chem. Int. Ed.* **2010**, 49, 2154-2157.

[24] J. Y. Liu, Z. X. Hu, Y. Z. Zhang, H. Y. Li, N. B. Gao, Z. L. Tian, L. C. Zhou, B. H. Zhang, J. Tang, J. B. Zhang, F. Yi, H. Liu, *Nano-Micro Lett.* **2020**, 12, 59.

[25] L. Song, J. Ahn, D.-H. Kim, H. Shin, I.-D. Kim, *ACS Appl. Mater. Interfaces* **2022**, 14, 28378-28388.

[26] X. Xin, Y. Zhang, X. X. Guan, J. X. Cao, W. L. Li, X. Long, X. Tan, *ACS Appl. Mater. Interfaces* **2019**, 11, 9438-9447.

[27] Y.-L. Zhang, C.-W. Jia, R.-N. Tian, H.-T. Guan, G. Chen, C.-J. Dong, *Rare Met.* **2021**, 40, 1578-1587.

[28] B. Liu, L. J. Zhang, Y. Y. Luo, L. Gao, G. T. Duan. *Small* **2021**, 17, 2105643.

[29] Z. Q. Li, M. Y. Qi, C. Y. Tu, W. P. Wang, J. R. Chen, A.-J. Wang, *Appl. Surf. Sci.* **2017**, 425, 765-775.

[30] Y. Jia, X. P. Huang, Z. F. Cao, S. Wang, H. Zhong, *Appl. Surf. Sci.* **2019**, 484, 864-875.

[31] C. Balamurugan, K. Cho, B. Park, J. Kim, N. Kim, Y. Pak, J. Kong, S. Kwon, *Chem. Eng. J.* **2022**, 430, 132690.

[32] F. B. Gua, Y. Z. Cuia, D. M. Han, S. Hong, M. Flytzani-Stephanopoulos, Z. H. Wang, *Appl. Catal. B-Environ.* **2019**, 256, 117809.

[33] L. L. Sui, X. F. Zhang, X. L. Cheng, P. Wang, Y. M. Xu, S. Gao, H. Zhao, L. H. Huo, *ACS Appl. Mater. Interfaces* **2017**, 9, 1661-1670.

[34] L. B. Deng, X. H. Ding, D. W. Zeng, S. P. Zhang, C. S. Xie, *IEEE Sens. J.* **2012**, 12, 2209-2214.

[35] Z. M. Cao, W. Wang, H. Ma, L. Xiao, J. Y. Li, Y. J. Sun, J. P. Sheng, F. Dong, *Sensor. Actuat. B-Chem.* **2022**, 353, 131155.

[36] D. Wang, Y. Yin, P. C. Xu, F. Wang, P. Wang, J. C. Xu, X. Y. Wang, X. X. Li, *J. Mater. Chem. A* **2020**, 8, 11188.

[37] L. Wang, S. Y. Song, B. Hong, J. C. Xu, Y. B. Han, H. X. Jin, D. F. Jin, J. Li, Y. T. Yang, X. L. Peng, H. L. Ge, X. Q. Wang, *Mater. Res. Bull.* **2021**, 140, 111329.

[38] A. Hermawan, B. Zhang, A. Taufik, Y. Asakura, T. Hasegawa, J. F. Zhu, P. Shi, S. Yin, *ACS Appl. Nano Mater.* **2020**, 3, 4755-4766.

[39] H. Chen, S. Ao, G.-D. Li, Q. Gao, X. X. Zou, C. D. We, *Sensor. Actuat. B-Chem.* **2019**, 282, 331-338.

[40] Y. J. Kwon, H. G. Na, S. Y. Kang, S.-W. Choi, S. S. Kim, H. W. Kim, *Sensor. Actuat. B-Chem.* **2016**, 227, 157-168.

[41] L. L. Wang, T. T. Zhou, R. Zhang, Z. Lou, J. N. Deng, T. Zhang, *Sensor. Actuat. B-Chem.* **2016**, 227, 448-455.

[42] H. Zhang, J. H. Hu, M. W. Li, Z. H. Li, Y. Yuan, X. L. Yang, L. L. Guo, *Sensor. Actuat. B-Chem.* **2021**, 349, 130734.

[43] Y. L. Zhang, C. W. Jia, Q. Y. Wang, Q. Kong, G. Chen, H. T. Guan, C. J. Dong, *Nanomaterials* **2019**, 9, 1059.

[44] Y. L. Zhang, C. W. Jia, Q. Y. Wang, Q. Kong, G. Chen, H. T. Guan, C. J. Dong, *Ind. Eng. Chem. Res.* **2019**, 58, 9450-9457.

[45] R. Zhang, S. Gao, T. T. Zhou, J. C. Tu, T. Zhang, *Appl. Surf. Sci.* **2020**, 503, 144167.

[46] H. Y. Liu, Y. H. He, K. Nagashima, G. Meng, T. T. Dai, B. Tong, Z. H. Deng, S. M. Wang, N. W. Zhu, T. Yanagida, X. D. Fang, *Sensor. Actuat. B-Chem.* **2019**, 293, 342-349.

[47] Y. Z. Zou, H. Wang, R. S. Yang, X. Y. Lai, J. W. Wan, G. Lin, D. Liu, *Sensor. Actuat. B-Chem.* **2019**, 280, 227-234.

[48] Z. M. Cao, Y. Z. Ge, W. Wang, J. P. Sheng, Z. J. Zhang, J. Y. Li, Y. J. Sun, F. Dong, *ACS Sens*. **2022**, 7, 1757-1765.

[49] M. L. Xiao, X. L. Yu, Y. C. Guo, M. F. Ge, *Environ. Sci. Technol.* **2022**, 56, 1376-1385.

[50] Z. W. Wang, P. J. Ma, K. Zheng, C. Wang, Y. X. Liu, H. X. Dai, C. C. Wang, H.-C. Hsi, J. G. Deng, *Appl. Catal. B-Environ.* **2020**, 274, 118963.
